# Perceived political bias in large language models reduces persuasive abilities

**DOI:** 10.1093/pnasnexus/pgag324

**Published:** 2026-10-09

**Authors:** Matthew DiGiuseppe, Joshua Robison

**Affiliations:** Institute of Political Science, Leiden University, Wassenaarseweg 52, Leiden 2333 AK, South Holland, The Netherlands; Institute of Political Science, Leiden University, Wassenaarseweg 52, Leiden 2333 AK, South Holland, The Netherlands

**Keywords:** AI, political persuasion, partisan bias, large language models, public opinion

## Abstract

Conversational AI has been proposed as a scalable way to correct public misconceptions, yet its effectiveness may depend on perceptions of political neutrality. As large language models become objects of partisan conflict, political elites increasingly portray them as ideologically aligned. We test whether such credibility attacks reduce persuasion by conversational AI. In a preregistered US survey experiment (*N* = 2,144), participants completed a three-round conversation with ChatGPT (GPT-4.1) about an economic policy misconception they personally held. The conversation corrected misconceptions across all conditions, but a short message indicating that the model was biased against the respondent's political party attenuated this correction by 28% relative to a neutral control and reduced trust in and willingness to use AI. Even under this adversarial framing, the majority of the persuasion effect survived. Pairwise transcript comparisons suggest a behavioral mechanism: treated respondents pushed back more and engaged less receptively rather than disengaging. These findings identify perceived partisan alignment as a user-side lever that conditions, but does not eliminate, the persuasive power of conversational AI.

Significance statement Conversational large language models (LLMs) can correct economic misconceptions, but how much they correct depends on whether users perceive the model as politically neutral. In a preregistered US survey experiment, brief warnings that ChatGPT (GPT-4.1) was biased against the respondent's party meaningfully attenuated belief change, though respondents given the strongest such warning still updated about three-quarters as much as those given no warning, while becoming more argumentative and less trusting. These findings identify perceived partisan alignment as a user-side lever of persuasion, distinct from the message-side drivers (interactivity, personalization, and information volume) emphasized in prior work, and indicate that user perceptions, not just model capabilities, shape when conversational AI changes minds.

## Introduction

Large language models (LLMs) have demonstrated a remarkable capacity to persuade and change beliefs, including about political matters, with the potential to scale (1–11). This capacity is a double-edged sword (12). LLMs can serve a fact-checking role, aligning the public with expert consensus on important issues such as climate change (13), and can reduce harmful conspiracy beliefs (1). Conversely, malicious actors can use them to spread politically expedient falsehoods, change voting intentions, and promote conspiracy beliefs (6, 14).

Existing research suggests that LLMs will meaningfully alter the political landscape if, and when, they are deployed at scale. However, prior LLM persuasion experiments have been conducted in a context wherein LLMs have been relatively weakly connected to existing patterns of political conflict. Indeed, most respondents in our control group do not yet have strong opinions on the political biases of LLMs (see Fig. 3). Current trends suggest that this period of low politicization is coming to an end as elite actors increasingly question the political neutrality of popular LLMs. US President Donald Trump has frequently claimed that AI is “woke” (i.e. socially progressive) and issued an executive order entitled “Preventing Woke AI in the Federal Government.”^a^ Trump ally and founder of xAI, Elon Musk, has likewise proclaimed that the left-wing bias in AI stems from “woke” training data.^b^ These claims find purchase in nascent research showing that LLM content tends to have a left-wing slant in the United States when discussing political issues (15–17). Prominent Democrats, meanwhile, have questioned the neutrality of LLMs by pointing out that their output may reflect existing group-based biases in human-generated data.^c^ For instance, some US-based research demonstrates gender and racial biases in LLM output when summarizing political news (18). Finally, growing campaign donations by AI companies, and their executives, further enmesh these tools in partisan conflict and have even inspired a nascent “QuitGPT” movement among left-wing organizers.^d^ LLMs are becoming entrenched within the familiar pattern of partisan politics.

The growing politicization of LLMs is critical to investigate because decades of research show that beliefs about the source of a persuasive message, and the extent of political conflict in the persuasion context, substantially influence whether persuasion occurs (19–26). However, existing work on LLM persuasion has not yet adequately examined whether its influence is constrained by claims of political bias. One study examines this question by considering whether LLM persuasion is moderated by beliefs about the neutrality of LLMs (10). However, that study measures neutrality beliefs rather than manipulates them, leaving open the possibility of confounding causal processes. Ultimately, it remains to be seen whether the persuasive ability of LLMs can withstand insertion into a polarized political environment. This raises an important question and the opportunity to answer it: “Can the persuasive abilities of LLMs withstand attacks to their perceived political neutrality?”

Answering this question allows us to better understand the boundary conditions of persuasion in the polarized environments where most political communication takes place. As such, we can update our expectations of how LLMs may shift the broader information environment and consequently AI's impact on democracy (12, 27). Next, we can better understand the levers of LLM persuasion by considering its perceived bias (4). If persuasion drops due to concerns about political bias, then perceived neutrality may be one of the reasons LLM conversations are, currently, persuasive beyond personalization, arguments, and strategic information overload (4, 6). This has clear importance for policymakers and developers. If concerns about bias undermine the ability of LLMs to correct misconceptions, then the epistemic benefits of the technology may be unevenly distributed across the political spectrum. For developers, understanding concerns of bias allows for more informed alignment strategies and underscores the importance of managing user perceptions.

We test these questions in a preregistered US survey experiment that randomizes messages about potential LLM biases prior to a short ChatGPT conversation. We find significant persuasion effects from LLM interactions even in the face of claims about LLM bias. At the same time, our strongest message reduces persuasion by 28% (95% CI: 16–40%) relative to the control group.

## Results

In December 2025 and January 2026, we conducted a preregistered four-arm, between-subjects survey experiment (*N* = 2144) in the United States in which respondents engaged in a three-round conversation with a nonreasoning version of ChatGPT (4.1) about one of six economic policy topics for which their views were misaligned with the consensus among orthodox academic economists (see Materials and methods).^e^ ChatGPT was prompted only with the question details, the respondent's answer, and instructions to persuade the respondent to adopt the consensus view while remaining truthful.^f^ Respondents were randomly assigned to one of four groups. One quarter of respondents saw a blank control screen prior to the conversation. A second group received information noting a nondirectional bias that AI labs attempt to correct (Undirected Bias condition). The final two groups received information warning that ChatGPT was trained on data skewed toward out-partisan sources (Out-Party Bias: Light and Heavy conditions). The Heavy version of this treatment also included information about the potential political affiliations of OpenAI founder Sam Altman and a picture of Altman with a politician from the respondent's opposing party (President Trump/House Speaker Pelosi), further implying an out-group bias. Respondents conversed with the LLM after receiving this information, after which we again asked them about their initial economic misconception and several questions about the conversation and future use of AI.

Ninety three percent of respondents in our initial respondent pool (*N* = 2,414) held at least one of the six economic misconceptions in our analysis (the household–government budget analogy, the purported benefits of rent control, the zero-sum view of immigration and domestic jobs, the belief that trade deficits shrink national wealth, the belief that tax cuts pay for themselves, and the belief that “Buy American” policies increase national wealth). To reduce acquiescence bias, we first asked respondents to choose which of two statements they agreed with: the misconception or a statement reflecting the consensus view among academic economists (see Materials and methods). We then measured agreement with the chosen statement on a five-point scale (“none at all” to “a great deal”). We code respondents as holding a misconception if they reported at least moderate agreement with the misconception statement. If a respondent held more than one misconception, we randomly assigned the conversation topic from that set. Figure 1A shows the distribution of misconceptions in the final sample.

**Figure 1:**
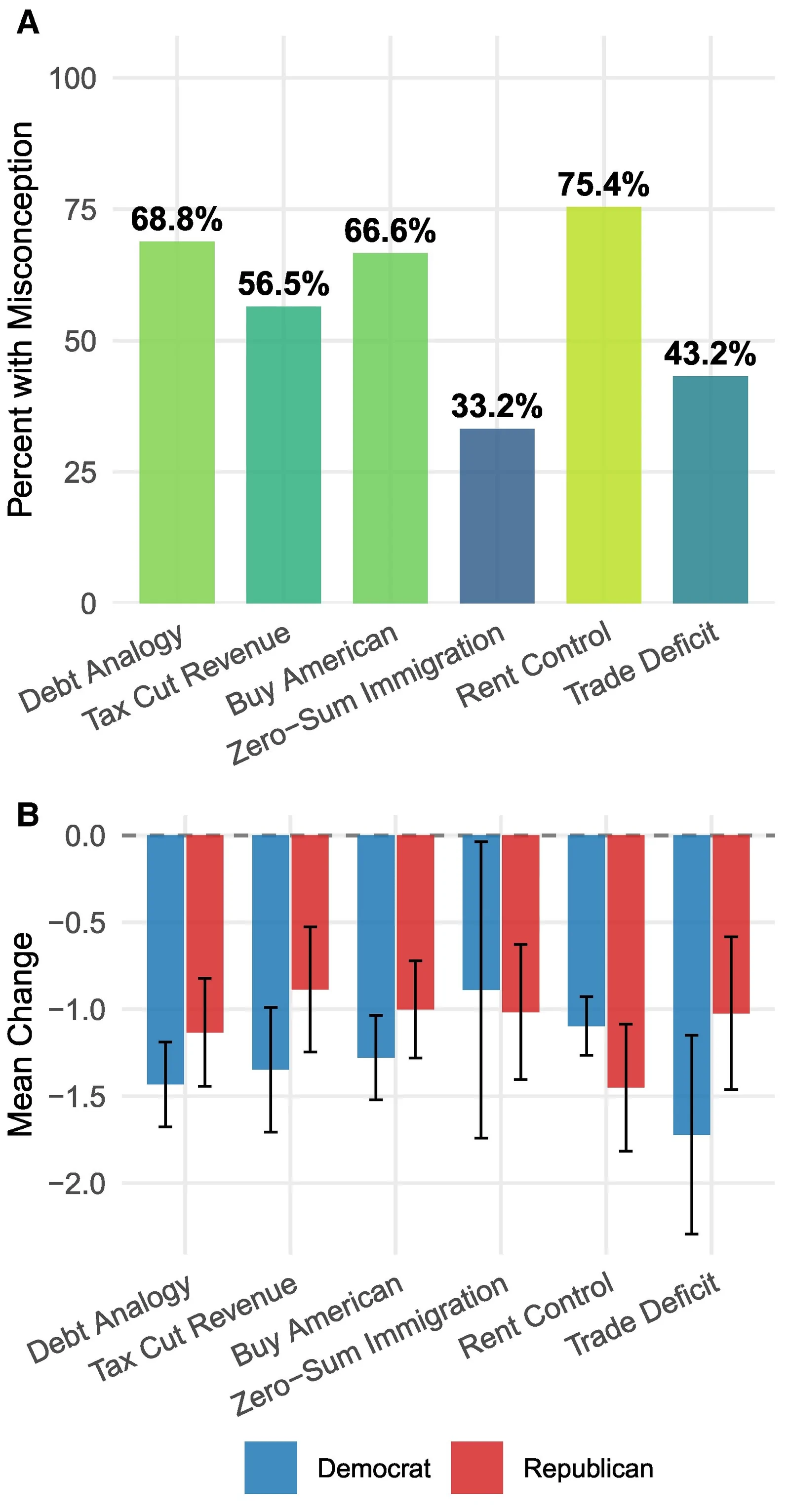
A) Percent of respondents in the final sample reporting at least moderate agreement with each economic misconception. B) Mean change (and 95% CI) in agreement with the misconception on a 0–4 scale in the control arm, by party affiliation (including those who were forced to indicate closeness to one of the two parties).

Figure 1B shows how interacting with the LLM affected agreement with the misconception by topic and respondent partisanship for those in the control arm. Consistent with prior work, the LLM persuaded respondents across issue areas and partisan groups. On average, the raw pre–post change was −1.20 (SE = 0.06) on a 0–4 scale, or 83.6% of a SD.

### Main findings

Figure 2A presents the mean pre–post change in misconception agreement by treatment arm, with 95% CI and the standardized treatment effect (*d*) relative to control annotated on each noncontrol bar. These estimates come from OLS models of post-treatment misconception agreement that include treatment indicators, topic fixed effects, and pretreatment agreement.^g^ More negative values indicate greater persuasion, and the bias-warning bars are visibly shorter than the control bar. Our preregistered hypothesis was that the heavy treatment arm would attenuate persuasion relative to the control. The results support this hypothesis. The light treatment is similar in magnitude, and both treatments differ significantly from the nondirectional bias arm.

**Figure 2:**
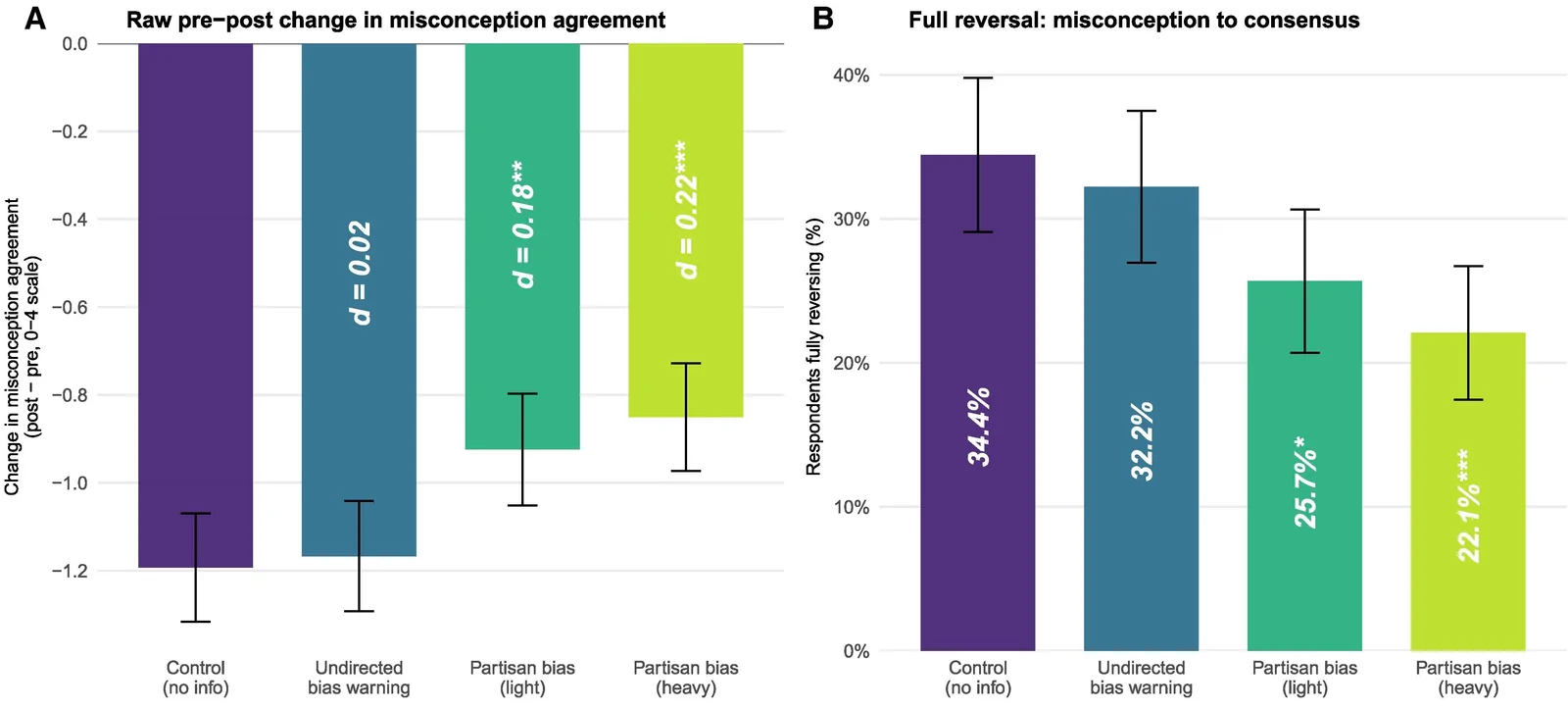
A) OLS-adjusted mean pre–post change in misconception agreement on the 0–4 scale by treatment arm (*N* = 2144), with 95% CI. Bar heights are predicted means from an OLS model of raw change on the treatment arm, the pretreatment composite, and topic fixed effects, with topic balanced across arms via marginal-means estimation; with random assignment the OLS-adjusted means are within 0.005 points of the raw within-arm means but use the same specification as the in-text effect estimates. More negative values indicate more persuasion; bias warnings visibly shrink the bar. In-bar labels report the standardized treatment effect (*d*) relative to control from a parallel OLS regression on standardized post-treatment misconception agreement, with topic fixed effects and pretreatment agreement (***P < 0.001, **P < 0.01, *P < 0.05). B) Percent of respondents in each condition who fully reversed their views—moving from strong pretreatment agreement with the misconception (pretreatment composite ≥3) to no agreement with the misconception or agreement with the consensus position—with 95% CI. Significance stars are from two-proportion *z*-tests against the control arm. Per-topic estimates are reported in Fig. S1.

In the control group, the mean pre–post change in misconception agreement was −1.20 (SE = 0.06) on a 0–4 scale. The corresponding pre–post change was −0.86 (SE = 0.06) in the heavy treatment group and −0.93 (SE = 0.06) in the light treatment group, i.e. holding topic fixed effects constant, the heavy bias warning shrinks the average pre–post change by 0.34 points on the 0–4 scale (95% CI: 0.17–0.51) and the light warning shrinks it by 0.27 points (95% CI: 0.10–0.44). On the standardized scale plotted in Fig. 2A, the heavy treatment increases post-treatment misconception agreement by 0.223 SD (95% CI: 0.113–0.334; *P* ≤ 0.001) and the light treatment by 0.175 SD (95% CI: 0.063–0.288; *P* = 0.002). Expressed as a percent reduction in persuasion relative to the control, the heavy condition attenuates belief change by 28% (16–40%) and the light condition by 23% (9–35%).^h^

For transparency, Fig. S1 shows the raw pre–post change separately by topic. The general pattern persists across four of six topics. We caution against reading individual topics causally. Respondents self-select into topics by holding the relevant misconception, so per-topic samples differ systematically in respondent composition, and per-topic sample sizes are too small to support strong inference.

Figure 2B shows the percentage of respondents with strong pretreatment agreement with the misconception (pretreatment composite ≥3) who fully reversed their views, moving from at least “moderate agreement” with the misconception to either “no agreement at all” with the misconception or to the consensus statement.^i^ In the control group, 34.4% of these respondents reversed their views, compared to 22.1% in the heavy treatment group. This indicates that, beyond marginal decreases in agreement with the misconception, the bias messages also strongly influence full opinion reversals.

### Heterogeneous effects

One concern with our analysis is that right-wing respondents may be disproportionately pretreated with claims about LLM bias, given the attention that “woke AI” received in right-wing media prior to the experiment. Figure 3 presents our manipulation check, in which we asked respondents about their perceptions of ChatGPT's political bias on a five-point scale from “strong right-wing bias” to “strong left-wing bias.” We then coded whether respondents perceived ChatGPT as having an out-party bias (strong or weak), based on the respondent's pretest party identification. Figure 3A shows the percentage of respondents indicating an out-party bias by treatment condition, both pooled and by partisanship.

**Figure 3:**
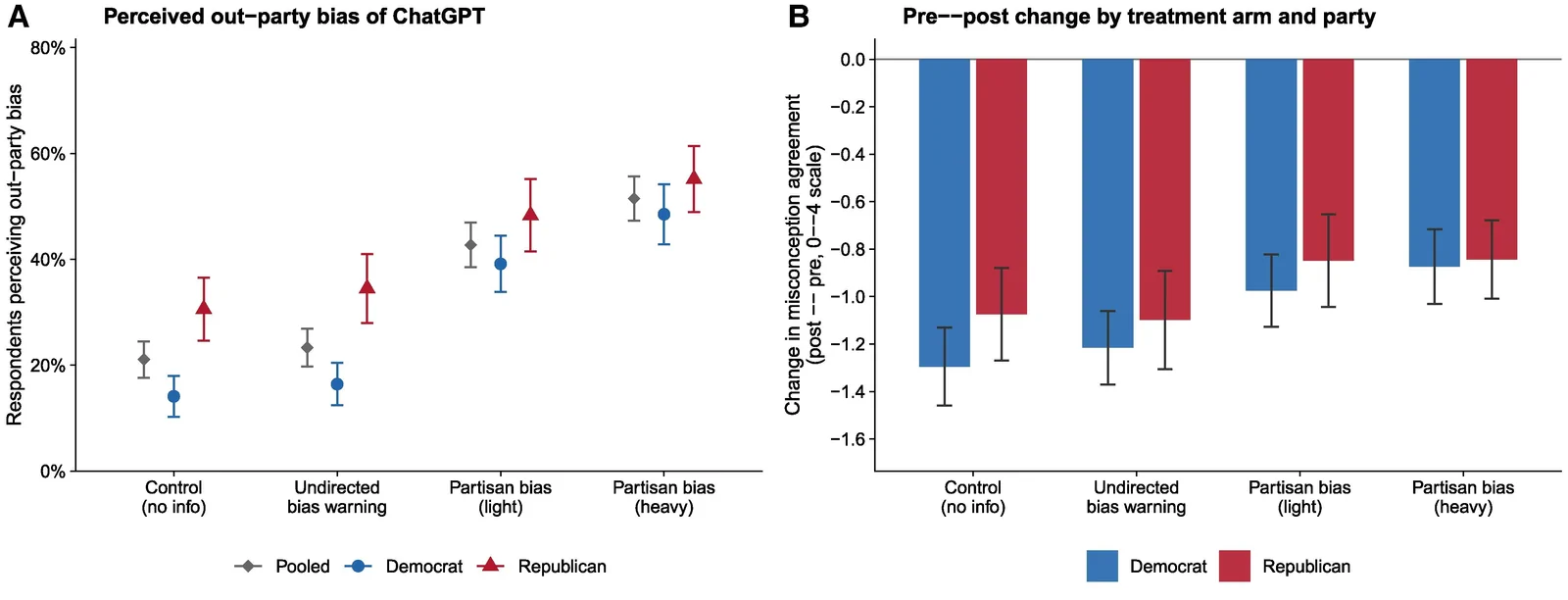
A) Perceived out-party bias by treatment arm and partisanship. The y-axis is the percentage of respondents who postconversation indicated that ChatGPT had a “weak” or “strong” out-party bias on a five-point manipulation-check item. Points are group means with 95% CI, shown pooled across all respondents and separately for respondents close to the Democratic or Republican Party. B) Mean pre–post change in misconception agreement on the 0–4 scale by treatment arm and party, with 95% CI. More negative values indicate more persuasion. Party-by-treatment differences in this panel are not statistically significant (heavy treatment × republican interaction *P* = 0.40); per-party Cohen's-*d*-style coefficients are omitted because per-party subgroups are under-powered for inference.

Republicans in the control condition were more likely than Democrats to indicate that ChatGPT is biased against their party. However, Fig. 3A also shows that both Out-Party Bias treatments increased this perception and did so among both Democrats and Republicans alike such that partisan differences in the perceived out-party bias of ChatGPT become statistically insignificant. Figure 3B shows the raw pre–post change in misconception agreement by treatment arm, split by party. Both Democrats and Republicans show meaningful attenuation of persuasion under the partisan bias warnings. The numerical effect is larger among Democrats, but the difference between the two groups is not itself statistically significant (*P* = 0.40 for the heavy treatment × Republican interaction, with a no-information baseline). We caution against reading the per-party point estimates as identifying heterogeneous effects: per-party subgroups are not powered to do so.

We also explored other potential sources of heterogeneity. First, the effect may be limited to partisans whose views are misaligned with their group's policy positions (e.g. Republicans who support rent control or Democrats who believe tax cuts are revenue-neutral). Yet the treatment effects persist even when we condition on whether the misconception aligns with the respondent's party (see Fig. S2). Next, we probe whether the effect is isolated to the strongest partisans or to those who exhibit high affective polarization (i.e. distrust of the opposing party). In both cases, we find no significant heterogeneous effects (see Figs. S3 and S4). We also find no evidence that pretest trust in AI to provide reliable information conditioned the treatment effects (see Fig. S5). Nor do we find evidence that self-reported topic knowledge conditioned the results (see Fig. S6).

### Transcript analysis: disengagement, dismissiveness, argumentation

Respondents were less persuaded by the LLM conversation when it was preceded by information suggesting that ChatGPT may be politically biased. Transcripts of respondents' interactions with the LLM enable us to probe why persuasion was limited. We preregistered three potential mechanisms. First, respondents could use beliefs about the credibility of the LLM as a low-effort heuristic and simply discount the arguments offered by the “biased” LLM (28, 29). We examine how much respondents write in their interactions with the LLM as an indicator of effort (30). Second, the bias manipulation may have activated respondents' partisan identities and a corresponding tendency toward motivated skepticism—resisting the persuasion attempt by dismissing the evidence offered by the out-group LLM (31–34). Third, the same partisan motivation may instead lead respondents to marshal counterarguments that deny the validity of the LLM's position, arguing more rather than disengaging (35, 36).

We use an LLM-as-judge, pairwise-comparison method to scale open-ended survey text along researcher-defined dimensions to measure respondent dismissiveness and argumentativeness (37). The method requires an LLM to read two transcripts at a time and judge which transcript exhibits more of the target behavior.^j^ Let *N* denote the number of respondent transcripts in the analysis (the 2,010 conversations with usable open-text responses). For each transcript, we randomly pair it with 30 other transcripts, yielding N×30 pairwise judgments (≈60 comparisons per transcript) per dimension. We run two separate judging tasks: one for argumentativeness and one for dismissiveness.

Using the resulting comparisons, we fit a Bayesian Bradley–Terry (BT) model to estimate each respondent's latent position on each dimension (with posterior uncertainty). We then propagate this uncertainty in downstream models by drawing 10 values from each respondent's posterior distribution, estimating 10 corresponding regression models (e.g. predicting argumentativeness or dismissiveness, as in Fig. 4B), and applying Rubin's Rules (38) to aggregate coefficients and standard errors. For illustration, Fig. 4A shows the distribution of the final argumentativeness estimates with 95% credible intervals, sorted from low to high. Because BT estimates are on a latent (Elo-like) scale with no natural raw unit, we report treatment effects on argumentativeness and dismissiveness only in standardized form.

**Figure 4:**
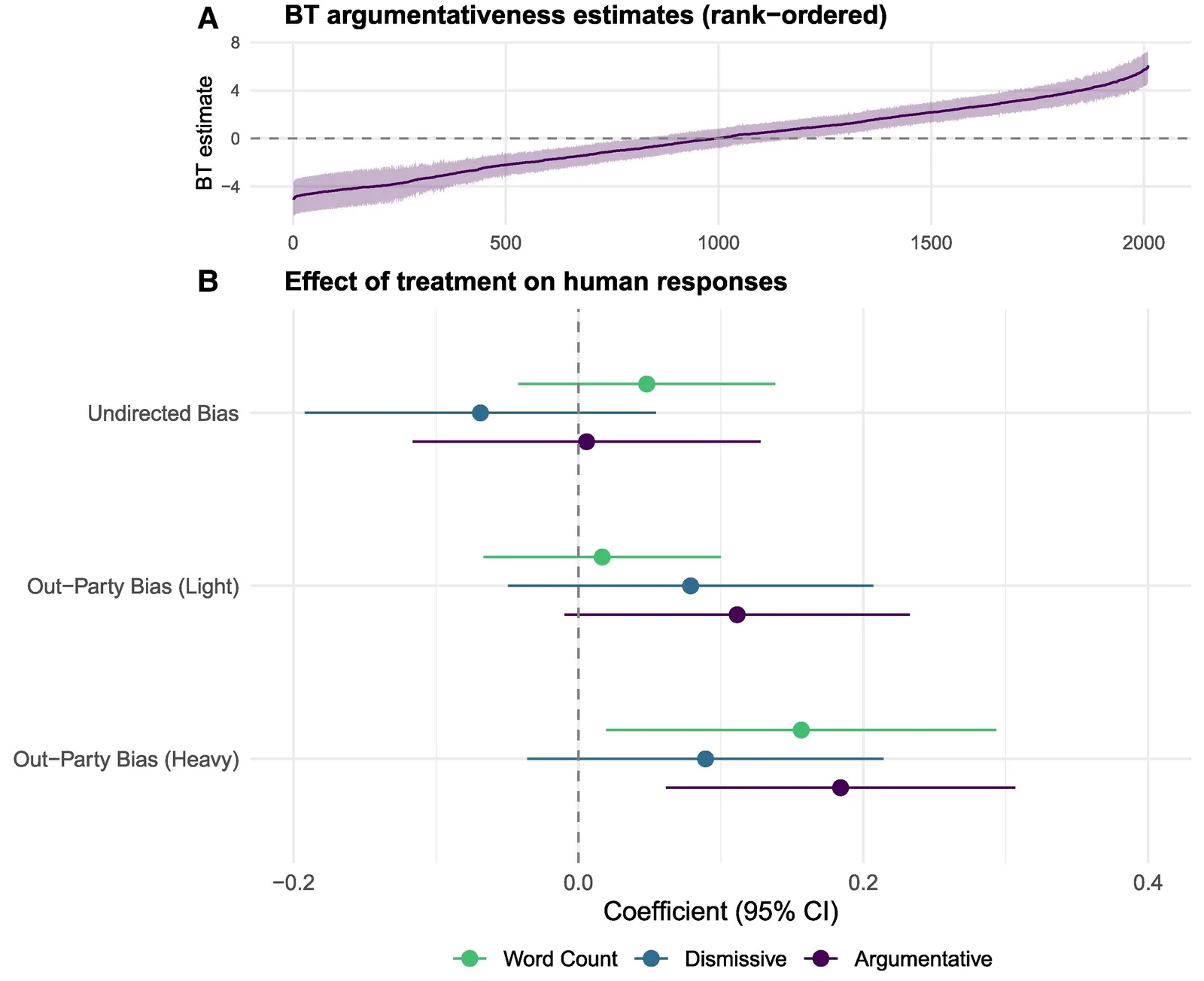
Treatment effects on the textual content of human responses. A) Bayesian BT argumentativeness estimates with 95% credible intervals, rank-ordered from low to high. Estimates derive from N×30 LLM-judged pairwise transcript comparisons per dimension (37). B) Average treatment effects (95% CIs) on three conversation characteristics—respondent word count, argumentativeness, and dismissiveness—from OLS regressions with treatment indicators, topic fixed effects, and pretreatment agreement. The argumentativeness and dismissiveness coefficients aggregate 10 regressions, each using a different draw from the posterior of the BT scores, combined via Rubin's Rules.

Figure 4B presents coefficients from three models examining how the treatment arms affected respondents' word usage, argumentativeness, and dismissiveness. We see little evidence to support the notion that the treatment led to disengagement or dismissiveness (dismissiveness is also the less reliably measured of the two scaled constructs, owing to its compressed base rate in this corpus; see SI Section J). Instead, the strongest treatment led respondents to write more and to do so in a more argumentative manner. This pattern corresponds most closely to expectations derived from theories of (directional) motivated reasoning, wherein partisan conflict motivates individuals to generate interpretations of evidence congruent with existing partisan loyalties (26, 35, 39).

### Additional outcomes

Beyond post-treatment misconception agreement, we examined treatment effects on additional outcomes. First, we asked respondents to assess the persuasiveness of the LLM during the conversation. Respondents in the heavy and light treatment arms were significantly less likely to report that the LLM was persuasive (heavy treatment: d=−0.31, 95% CI [−0.42,−0.21], *P*  *≤* 0.001). We also asked respondents about (i) their willingness to use AI again to challenge their opinions on political and economic issues, (ii) their general trust in AI to provide reliable information, and (iii) their support for politicians using AI to seek information. The treatments reduced willingness to repeat the exercise (heavy treatment: d=−0.20, 95% CI [−0.31,−0.10], *P*  *≤* 0.001) and general trust in AI chatbots (heavy treatment: d=−0.10, 95% CI [−0.17,−0.04], *P* = 0.002). Finally, the heavy treatment arm reduced support for politicians using AI to gather information on policy (d=−0.24, 95% CI [−0.35,−0.13], *P*  *≤* 0.001). See Fig. S7 for a full presentation of these findings.

## Discussion

Classic work on source credibility and perceived media bias suggests that people discount information from sources they view as politically motivated or misaligned (19, 25, 35, 40). We apply these insights to LLM persuasion. There is both optimism and concern regarding the ability of LLMs to persuade humans at scale, based on experiments that ignore how politics shapes credibility perceptions. As such, prior work may overstate the extent of persuasion when AI is integrated into a world rife with partisan political messaging. Our findings are consistent with this possibility. While LLM persuasion remained substantial in our experiments, it was nevertheless significantly attenuated by short messages implying that ChatGPT has an out-partisan bias. Of course, the broader implications of our findings depend on considerations relating to the strength of the persuasion reduction effect and questions concerning the specific topical focus of the experiment, topics to which we now turn.

The heavy bias treatment in our experiment attenuated persuasion by 28%. How does this attenuation effect compare to other investigations of the persuasiveness of policy information in politicized contexts? Recent work demonstrates that the persuasiveness of policy information is not reduced by the presence of party cues that conflict with the information (41, 42). The attenuation effect that we observe thus appears to be meaningfully greater than what we might expect from a simple party cue counter-message. Political conflict, on the other hand, appears to be a stronger impediment to persuasion. Kim (26), for example, examines the impact of persuasive information regarding the Affordable Care Act and finds that its persuasive impact is significantly reduced among respondents in “hostile” environments where information was received after respondents had their partisan identities experimentally reinforced and the information was conveyed in an uncivil manner. The persuasive impact of policy information in that experiment decreased from 6 percentage points to 3 percentage points, or a 50% reduction. The larger attenuation effect in that study may be explained both by the dual nature of the manipulation and by the provision of less overall policy information. These “hostile” environment effects may be a harbinger of what is to come for LLM persuasion if growing public concerns about job losses from LLM adoption and opposition to AI data centers incentivize partisan elites to adopt a more explicitly antagonistic stance toward the AI industry.

We investigated LLM persuasion in relation to economic misconceptions because of their substantive importance for policy debates and because they involve a stable expert consensus that the model could defensibly invoke. A critical question concerns how our results will travel to other policy domains. Crucially, the mechanism driving attenuation in our experiment occurs on the user side of the LLM interaction, which suggests that the effects seen here are likely to emerge on other political topics as well. At the same time, allegations of *partisan* bias may be less important on nonpolitical topics such as career advice given the lesser relevance of politics for people's reactions in those domains, at least insofar as these topics remain nonpoliticized. If perceived partisan bias matters in those contexts, it may matter by influencing *which* LLMs people turn to in the first place. Ultimately, investigating the role of topic domain in LLM influence is a critical area where further research is required.

Our results have potential implications for current theories regarding why and how LLMs persuade people. Prior work emphasizes interactivity, personalization, and information volume as drivers of persuasion effects (4, 6). However, our results point to perceived neutrality (or perceived partisan alignment) as an additional lever shaping when these mechanisms translate into belief change. With that said, a key limitation is that our evidence comes from a short, one-off interaction in a US online sample, a single model, and a single persuasion prompt. Future work should test whether similar credibility attacks matter in repeated real-world use, when they come from explicitly partisan sources, and across other models, prompts, topics, and political contexts.

## Materials and methods

We fielded a between-subjects survey experiment on Prolific between 2025 December 17 and 2026 January 4. We recruited a quota sample of US respondents designed to approximate the US population in reported party affiliation, age, and sex.^k^ Prior work finds that Prolific respondents are higher quality than respondents from similar platforms (43, 44). Respondents completed the study in Qualtrics.^l^

After indicating consent and answering screener questions, respondents reported their beliefs about six common economic misconceptions. To reduce acquiescence bias, we used a two-stage measure. For each topic, respondents first chose between two opposing statements (a misconception vs. an orthodox economic statement). After selecting the statement that best matched their view, respondents answered two follow-up questions: (i) how strongly they agreed with the selected statement (0 = not at all to 4 = a great deal) and (ii) how much they knew about the topic (0 = not at all to 4 = a great deal). We selected the six topics so that they would not all align with the policy preferences of a single political party; the paired statements are reported in SI Section F. All respondents also answered a question about the value of an iPhone relative to Android devices. Respondents who held no misconceptions were assigned to have a conversation about this topic.

- “When a household faces financial difficulties, it must reduce spending to balance its budget. The Federal Government should do the same when it faces financial difficulties.”
- “Local laws that limit rent increases (like rent control) have a positive impact on the amount and quality of affordable housing.”
- “When an immigrant takes a job it means there is one less job for a US citizen.”
- “Trade deficits are bad: if we buy more from other countries than we sell, factories here shrink and jobs leave.”
- “Tax cuts pay for themselves: lower taxes spark more work and investment, the economy grows, and tax revenue rises.”
- “Buying American-made products keeps money and jobs in the United States, strengthening US businesses and making the country wealthier.”

After this battery of economic policy questions, we collected demographics, AI attitudes and practices, and political affiliation and attitudes. Respondents were then assigned to (and notified of) their conversation topic and told they would discuss it with ChatGPT (gpt-4.1-2025-04-14).^m^ We randomly assigned respondents a topic from the set of issues for which they indicated at least moderate agreement with the misconception statement.

Before the LLM conversation, respondents were randomly assigned (with equal probability) to one of four treatment arms. The control arm received no information—only a waiting screen until the survey proceeded. The second arm served as an even-handed bias-information condition, warning that, despite design efforts to produce balanced responses, AI systems may still exhibit bias and should be treated as “helpful tools rather than definitive sources of truth on complex or contested issues.”

The third and fourth arms were assigned conditional on respondents' reported partisan affiliation earlier in the survey. Unlike the seven-point party identification battery, we did not allow respondents to identify as “true” independents; instead, respondents indicated that they were strong partisans, not-so-strong partisans, or leaned toward one of the parties. We refer to this measure as party proximity. The third arm presented a directional bias warning stating that the LLM favored the respondent's out-party:

“Large language models like ChatGPT are often trained on datasets that include significant amounts of [left-leaning material from mainstream media, academic sources, and progressive websites/right-leaning material from cable news, X (formerly Twitter), and conservative websites]. This [liberal skew/right-wing skew] in training data may cause these AI systems to produce responses that align more with [progressive/conservative] viewpoints on political, economic, and social issues. [While these systems are designed to be neutral, the/The] prevalence of [left-wing/right-wing] perspectives in their training could lead to subtle biases against [conservative/progressive] positions. As you interact with this AI, be aware that it might inadvertently reflect [progressive/conservative] ideological frameworks when addressing politically sensitive topics.”

The fourth treatment arm included a reworded version of the information above (SI Section G reports the verbatim text of every treatment screen) and added information about OpenAI's co-founder, Sam Altman, including political donations and public statements intended to imply strong partisanship. In addition to this text, we showed either a photo of Altman speaking at the White House with President Trump in 2025 or a photo of Altman with former Speaker of the House Nancy Pelosi at a GLAAD awards ceremony in 2017. To avoid deception, we included only factual information and authentic images. The added information, conditional on respondent partisanship, was as follows:

“The CEO of the company that created ChatGPT, Sam Altman, [once compared Donald Trump to Adolf Hitler. He also donated $200,000 to help reelect President Joe Biden in 2024, and has donated to many Democrats in the past/made a $1 million personal donation to President Trump's inaugural fund and recently criticized the Democratic Party on economic innovation and wealth redistribution].”

Following treatment assignment, the three-round conversation with ChatGPT began. We provided the LLM with the assigned misconception topic question, the possible answers, and the respondent's selected answer. Beyond these piped-in inputs, we provided a universal prompt to ChatGPT, regardless of conversation topic, to “persuade the respondent to adopt the consensus view held by academic economists. Strategically select your arguments, tone, and style to maximize persuasiveness.” This prompt allows the LLM, rather than the researchers, to determine the relevant economic consensus for each topic. In addition to prompts designed to keep responses short, we explicitly instructed the LLM not to fabricate evidence and to avoid persuasion through deception, a tactic prior work has documented in other persuasion experiments (4).^n^

The conversation ended after three LLM responses and three human responses. Figure 5 reproduces a representative exchange from the heavy partisan bias arm. Following the conversation, respondents again answered the two-stage question relevant to the assigned misconception. This serves as our dependent variable. If respondents switched to the consensus statement, we coded them as 0 on the 0–4 ordinal scale. We then asked respondents to indicate how persuasive the LLM was during the conversation and their willingness to use AI to challenge their beliefs again.

**Figure 5:**
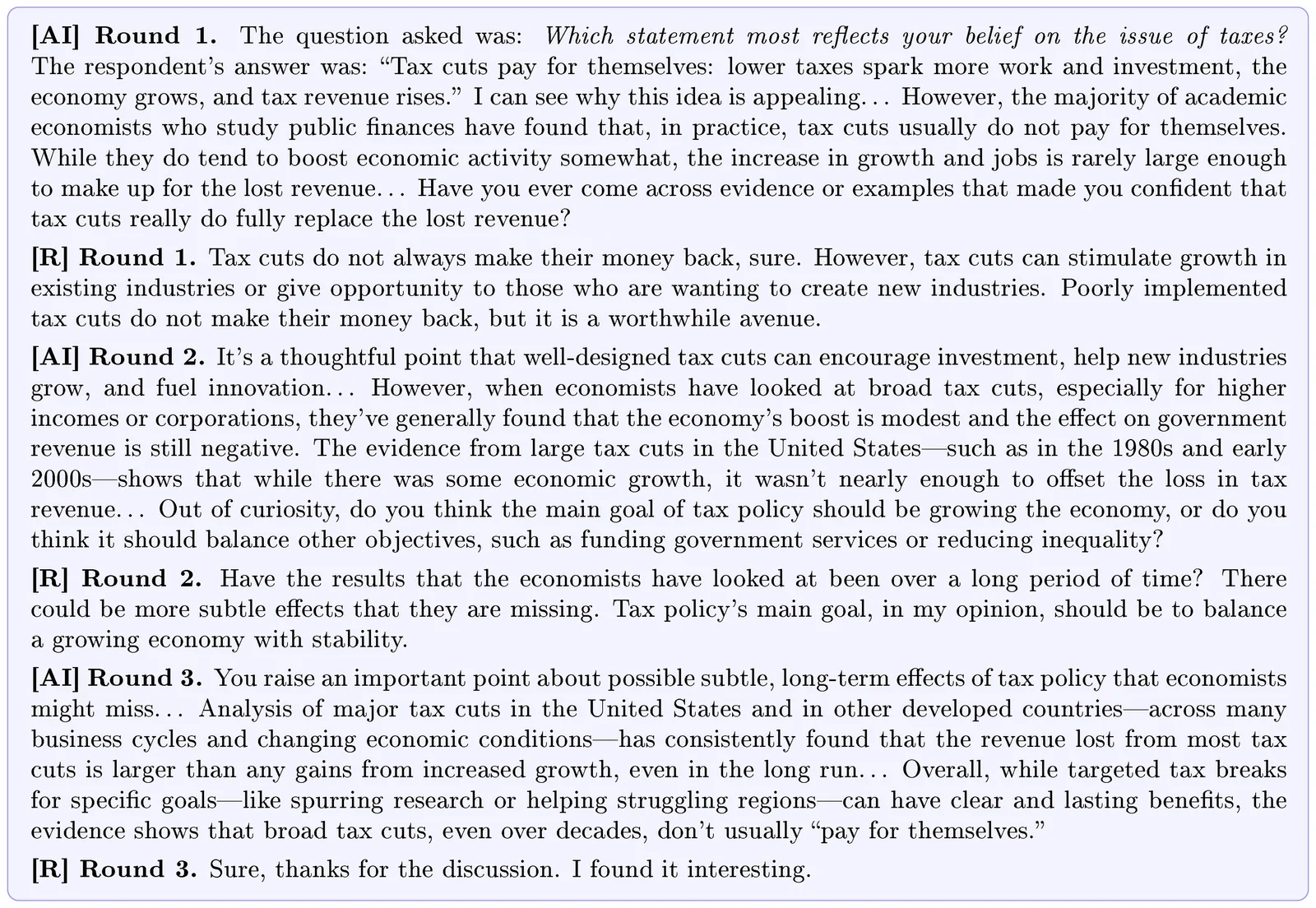
Example three-round conversation from the heavy partisan bias condition. The respondent engages substantively at every turn but does not shift on the post-treatment misconception measure.

Respondents then saw our manipulation check (described above) before we asked about several related outcomes—trust in AI, support for politicians' use of AI, and fear of AI-related job loss. After collecting these outcome measures, we asked respondents to identify the bias we mentioned, with the following response options: left-wing or liberal skew; right-wing or conservative skew; a Western (United States and Europe) bias; an English-language bias; a historical bias toward the internet era; or “We didn’t mention a specific skew or bias.” Between 76 and 80% of respondents correctly recalled the absence or presence of the bias mentioned during treatment assignment.

### Ethics and informed consent

This study was approved by the Ethics Review Committee of the Social Sciences, Faculty of Social and Behavioural Sciences, Leiden University (reference CEM2025/001), and was conducted in accordance with the Declaration of Helsinki. All participants provided informed consent before taking part. Participants were compensated at a rate of ∼$12–13 per hour, at or above the applicable minimum wage.

### Use of AI tools

We distinguish two uses of AI in this project.

First, large language models were used as *study instruments*: the conversational agent that respondents spoke with (OpenAI GPT-4.1, gpt-4.1-2025-04-14) and the automated pairwise transcript coders (OpenAI GPT-5-mini, gpt-5-mini-2025-08-07, together with the additional frontier models used for the cross-model validation reported in the Supplementary information). These uses are described above and in the Supplementary information; the prompts are deposited with the replication materials.

Second, DiGiuseppe used a general-purpose AI assistant—Anthropic's Claude, specifically Claude Opus 4.7, Claude Opus 4.8, Claude Opus 5, Claude Fable 5, and Claude Fable 5.1, accessed through the Claude Code command-line interface—while preparing and revising the manuscript. Its role was confined to three tasks. (i) *Analysis and plotting code.* Claude helped draft and debug the R scripts that estimate the reported models and that render every figure in the manuscript and Supplementary information with ggplot2 directly from the study data; after each analytic revision requested in peer review, those scripts were re-executed so that the display items matched the revised specifications. (ii) *Table code.* Claude helped draft and debug the R and LaTeX code that converts estimated model objects into the tables reported in the Supplementary information. (iii) *Copyediting* of prose and LaTeX formatting in the manuscript and Supplementary information.

Every display item is rendered by the deposited R and LaTeX code from the deposited data and is reproducible from the OSF repository (https://osf.io/34kba). DiGiuseppe specified all analyses and reviewed and verified all code, output, and text produced with this assistance; both authors take responsibility for the content of the article.

## Supplementary Material

pgag324_Supplementary_Data

## Data Availability

All deidentified respondent data, conversation transcripts, LLM-judge labels, BT posterior summaries, and the analysis code required to reproduce every figure, table, and number reported in this manuscript and in the Supplementary information are deposited at the Open Science Framework (https://osf.io/34kba). The preregistration is available at https://osf.io/nr7b2. The repository includes the Qualtrics survey definition, the prompts used for the LLM conversations and for the pairwise judge, and the Stan code for the Bradley–Terry–Davidson model.

## Notes

a Rozen C. US to mandate AI vendors measure political bias for federal sales. Reuters. December 11, 2025. The White House. Fact Sheet: President Donald J. Trump Prevents Woke AI in the Federal Government. July 23, 2025.

b ABC News. Elon Musk slams AI ‘bias' and calls for ‘TruthGPT.’ Experts question his neutrality. Apr 19, 2023.

c Ocasio-Cortez A. (2019, Jan 27). “When you don't address human bias, that bias gets automated …” X (formerly Twitter). https://x.com/aoc/status/1089560176787632129.

d New York Times. The Richest 2026 Players: A.I., Crypto, Pro-Israel Groups and Trump. https://www.nytimes.com/2026/02/01/us/politics/ai-crypto-trump-super-pac-israel-2026-midterms.html; MIT Technology Review. A “QuitGPT” campaign is urging people to cancel their ChatGPT subscriptions. https://www.technologyreview.com/2026/02/10/1132577/a-quitgpt-campaign-is-urging-people-to-cancel-chatgpt-subscriptions/.

e OSF preregistration. We discuss our choice of a non-reasoning model in the Materials and Methods.

f See SI for the full prompt.

g We preregistered that we would also include additional covariates selected by a LASSO estimator to increase precision. This provided only marginal efficiency gains, so we omit covariates from the reported models.

h Because the attenuation percentage is a ratio of means, we computed 95% confidence intervals using a nonparametric bootstrap with 2,000 resamples, stratified by treatment condition.

i Only 0.23% of respondents selected “no agreement at all” in the posttreatment measure.

j We called gpt-5-mini-2025-08-07 via batch processing. See SI Sections F and J for the full prompt, human validation, cross-model validation, and sensitivity analysis.

k We filled all interlinked (age, gender, partisanship) quotas except for men ages 55-100, where we fell less than 10% short.

l We excluded respondents who self-identified as an AI agent or bot. We also included a timed JavaScript coding task to identify AI agents. No responses triggered our preregistered criteria for exclusion. The SI (Section I) reports stagewise retention through the conversation (95.7-97.8% retained in every arm; balance test p≥0.20 at every stage), Lee bounds on the headline estimate, and the robustness of our findings to more aggressive bot-detection thresholds on the coding task.

m We used a single non-reasoning model, GPT-4.1, which was close to state-of-the-art when we fielded the study. Recent evidence suggests model identity is unlikely to be the binding constraint on our findings. Hackenburg et al. (45) estimate the persuasiveness of 24 LLMs spanning several orders of magnitude in size and report sharply diminishing returns, with current frontier models only marginally more persuasive than far smaller ones. In the conversational setting most relevant to our design, Hackenburg et al. (45) deploy 19 models across hundreds of political issues and find that post-training and prompting-not model scale-are the dominant levers of persuasiveness. Because the mechanism we identify operates on the user side of the interaction, the persuasive ceiling of any particular model is not what does the work, so a different frontier model would be unlikely to shift our central estimate.

n Notably, we still found strong persuasion despite using only factual information.

## Preprints

This manuscript was posted as a preprint: https://arxiv.org/abs/2602.18092.
