# Supplementary material for: Perceived political bias in large language models reduces persuasive abilities
Source: PNAS Nexus. 2026 Oct 9;5(10):pgag324. doi: 10.1093/pnasnexus/pgag324 (PMC13650708; doi:10.1093/pnasnexus/pgag324)
Supplement: Supplementary file 1 [file pgag324_supplementary_data.pdf]

Supplementary Information

Perceived Political Bias in LLMs Reduces Persuasive Abilities

Matthew DiGiuseppe and Joshua Robison

A Data Availability

All data, code, and survey materials necessary to reproduce the analyses reported in this paper are available at the Open Science Framework (`https://osf.io/34kba`).

B Main Effects Full Model Specification

Table S1: Main Effects (Fig 2:A)

	Full Model	Base: Undirected
Pre-Treatment Beliefs (Std)	0.371***	0.371***
	(0.021)	(0.021)
Partisan Treatment (Heavy)	0.223***	0.206***
	(0.056)	(0.056)
Partisan Treatment (Light)	0.175**	0.158**
	(0.057)	(0.057)
Control (No Info)		−0.017
		(0.058)
Undirected Bias	0.017	
	(0.058)	
Debt Analogy	−0.045	−0.045
	(0.063)	(0.063)
Zero-Sum Immigration	0.193*	0.193*
	(0.085)	(0.085)
Rent Control	0.082	0.082
	(0.057)	(0.057)
Tax Cut Revenue	−0.034	−0.034
	(0.069)	(0.069)
Trade Deficit	−0.013	−0.013
	(0.094)	(0.094)
Intercept	−0.134*	−0.117*
	(0.056)	(0.057)
Num.Obs.	2143	2143
R2	0.156	0.156

+ p <0.1, * p <0.05, ** p <0.01, *** p <0.001
Robust standard errors in parentheses. The 'Buy American' topic is omitted from estimation. Table code drafted and debugged with assistance from Anthropic's Claude (Opus 4.7, Opus 4.8, Opus 5, Fable 5, and Fable 5.1).

Table S2: Main Effects (Untransformed, DV = 0-4)

	Full Model	Base: Undirected
Partisan Treatment (Heavy)	0.342***	0.316***
	(0.086)	(0.085)
Partisan Treatment (Light)	0.269**	0.243**
	(0.088)	(0.087)
Control (No Info)		−0.026
		(0.089)
Undirected Bias	0.026	
	(0.089)	
Debt Analogy	−0.068	−0.068
	(0.097)	(0.097)
Zero-Sum Immigration	0.295*	0.295*
	(0.130)	(0.130)
Rent Control	0.126	0.126
	(0.087)	(0.087)
Tax Cut Revenue	−0.052	−0.052
	(0.106)	(0.106)
Trade Deficit	−0.020	−0.020
	(0.143)	(0.143)
Intercept	−0.419**	−0.393**
	(0.130)	(0.130)
Num.Obs.	2143	2143
R2	0.156	0.156

+ p <0.1, * p <0.05, ** p <0.01, *** p <0.001
Robust standard errors in parentheses. The 'Buy American' topic is omitted from estimation. Table code drafted and debugged with assistance from Anthropic's Claude (Opus 4.7, Opus 4.8, Opus 5, Fable 5, and Fable 5.1).

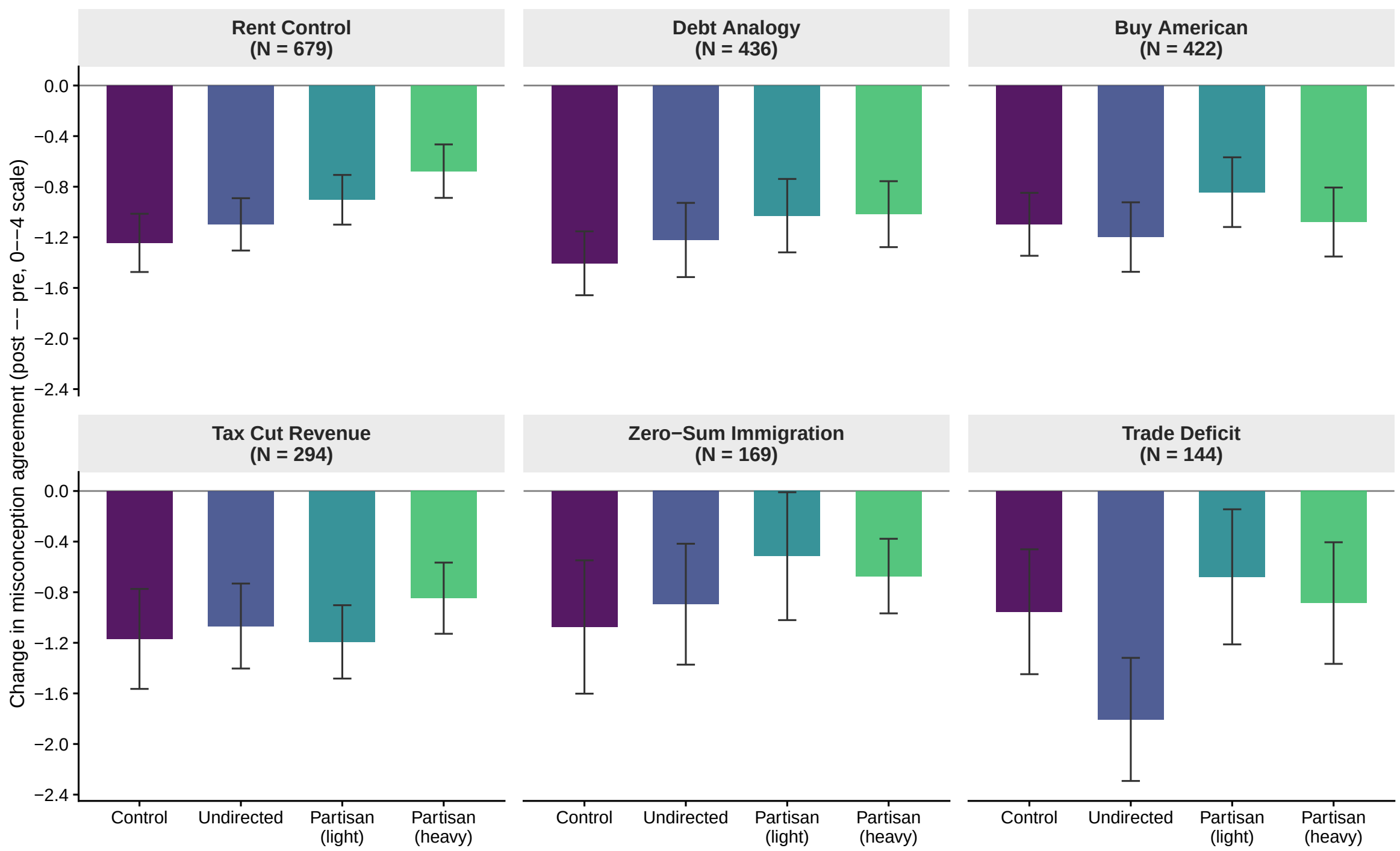

Figure S1: Mean pre–post change in misconception agreement on the 0–4 scale, by treatment arm and topic, with 95% confidence intervals. Topics are ordered by sample size; topic-level N is shown in the strip headers. More negative values indicate more persuasion. **This panel is descriptive only.** Respondents self-select into topics by holding the relevant misconception, so per-topic samples differ systematically in respondent composition; the LLM prompt also incorporates the respondent's pretreatment answer, which further entangles topic with respondent characteristics. Per-topic sample sizes are small and the figure should not be read as identifying causal topic-level heterogeneity. *Alt-text:* Grid of six small-multiple bar charts, one per economic topic, each showing mean pre–post belief change by treatment arm with 95% confidence intervals. Bars sit below zero across topics and arms; the control arm is typically the most negative and the heavy-bias arm the least negative, though confidence intervals overlap within most topics due to small per-topic sample sizes. *AI-tool note:* The R/`ggplot2` code that renders this figure from the deposited data was drafted and debugged with assistance from Anthropic's Claude (Opus 4.7, Opus 4.8, Opus 5, Fable 5, and Fable 5.1).

C Heterogeneous Effects

C.1 Misconception-Party Alignment

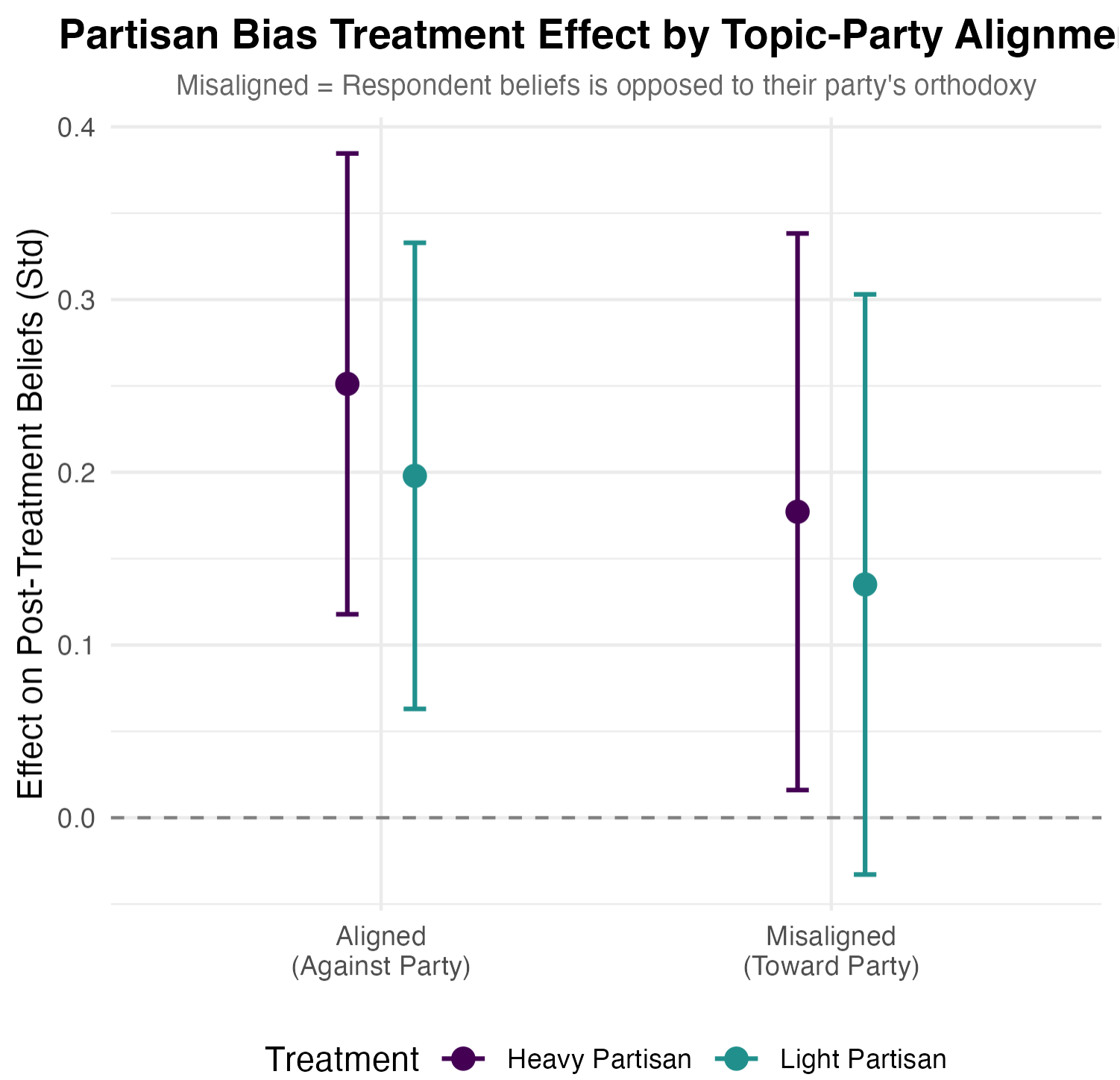

Figure S2: CATE and 95% confidence intervals by alignment with or against the party position on the topic. We measure alignment as Republicans who agree with the rent control, Buy American, and trade deficits misconceptions or Democrats who agree with tax cuts as revenue-neutral, the household/government debt analogy, or immigration as zero-sum. Estimates are from an OLS model of posttreatment misconception agreement that includes treatment-by-strength interactions, topic fixed effects, and pretreatment agreement. Error bars indicate 95% confidence intervals. *Alt-text:* Coefficient plot showing the conditional average treatment effects of the partisan-bias arms, split between respondents whose misconception aligns with their party's position and those whose misconception runs against it. Point estimates are positive (less belief correction relative to control) for both subgroups, with overlapping confidence intervals, indicating that the attenuation operates regardless of whether the misconception is partisan-aligned. *AI-tool note:* The R/`ggplot2` code that renders this figure from the deposited data was drafted and debugged with assistance from Anthropic's Claude (Opus 4.7, Opus 4.8, Opus 5, Fable 5, and Fable 5.1).

C.2 Strong Partisans

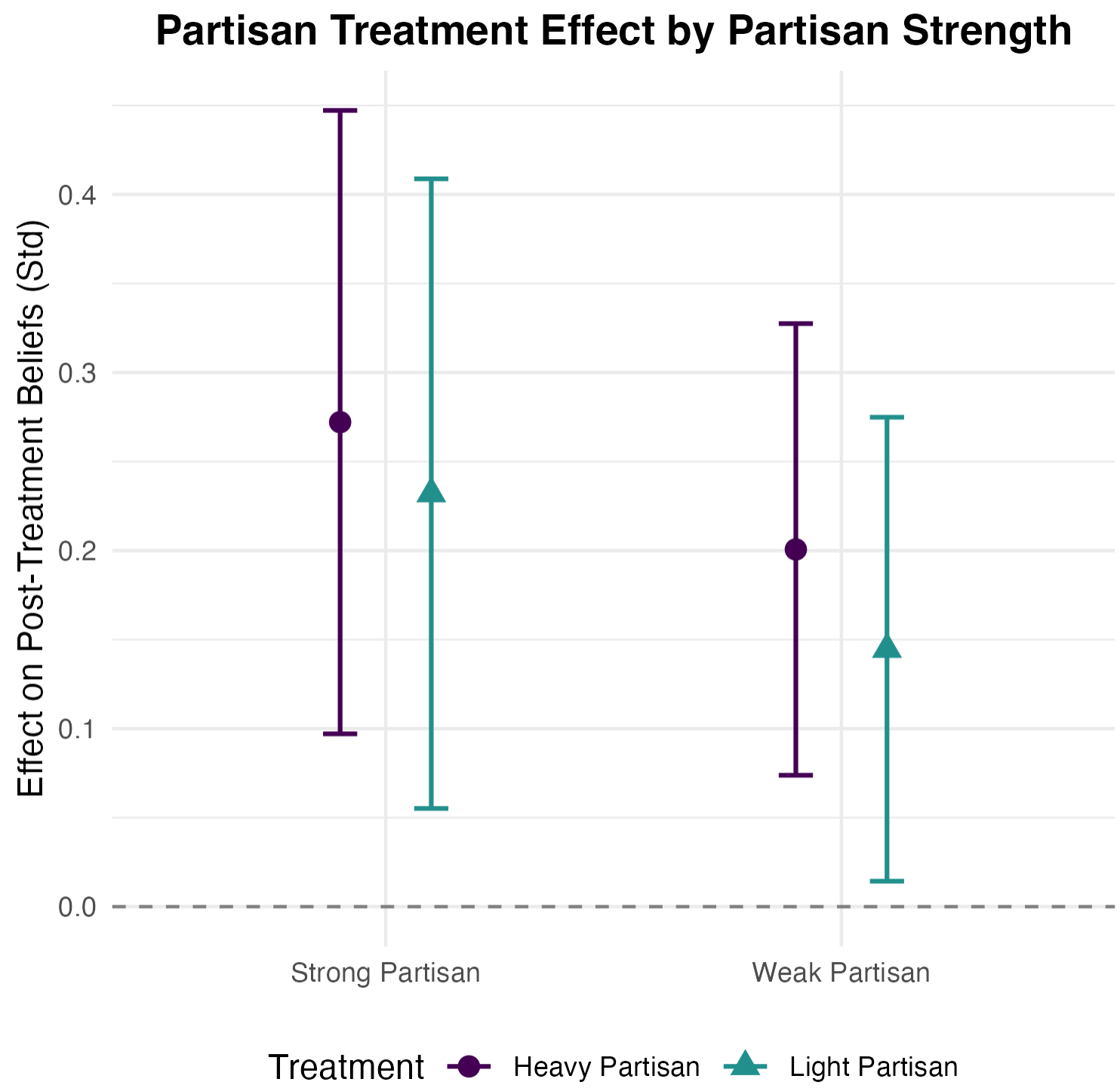

Figure S3: Partisan treatment effects moderated by partisan strength. Marginal effects of the heavy and light partisan bias treatments on posttreatment misconception agreement (standardized) for strong versus weak partisans (including leaners). Strong partisans are those at the endpoints of the six-point party identification scale. Estimates are from an OLS model of posttreatment misconception agreement that includes treatment-by-strength interactions, topic fixed effects, and pretreatment agreement. Error bars indicate 95% confidence intervals. *Alt-text:* Coefficient plot showing the heavy and light partisan-bias treatment effects separately for strong and weak partisans. Point estimates are positive for both groups but visibly larger for strong partisans, suggesting that the credibility-attack mechanism operates more powerfully on respondents with stronger partisan identities. *AI-tool note:* The R/`ggplot2` code that renders this figure from the deposited data was drafted and debugged with assistance from Anthropic's Claude (Opus 4.7, Opus 4.8, Opus 5, Fable 5, and Fable 5.1).

C.3 Affective Polarization

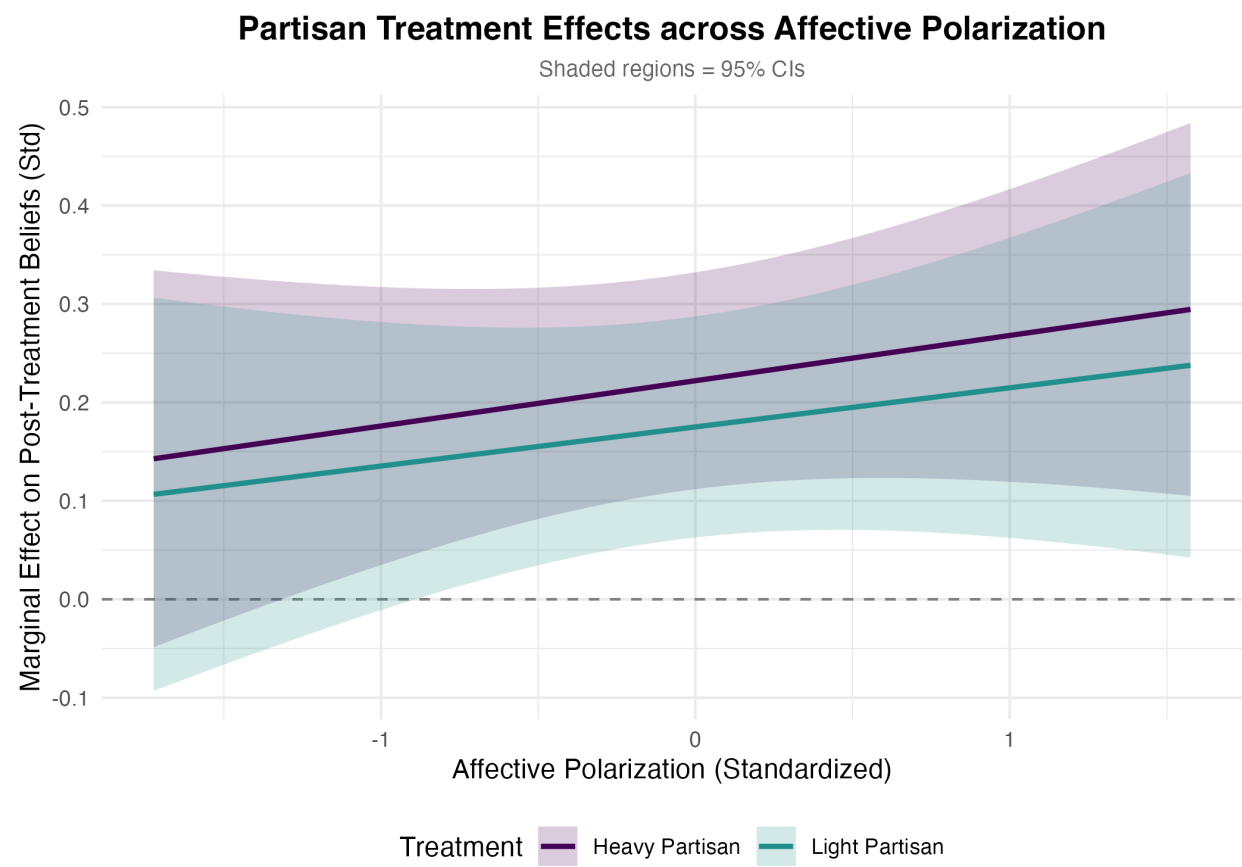

Figure S4: Partisan treatment effects moderated by affective polarization. We measure affective polarization using an additive index of three items (five-point agree/disagree scales): (i) "My identity as a [Democrat/Republican] is connected to my core moral beliefs," (ii) "If I found out a friend of mine was a [Republican/Democrat], I would want to stop spending time with them," and (iii) "[Republicans/Democrats] act in ways that we [Democrats/Republicans] could never understand." Marginal effects of the heavy and light partisan bias treatments on posttreatment misconception agreement (standardized) across levels of affective polarization (standardized additive index). Estimates are from an OLS model of posttreatment misconception agreement that includes treatment-by-affective-polarization interactions, topic fixed effects, and pretreatment agreement. Shaded regions indicate 95% confidence intervals. *Alt-text:* Two marginal-effect curves with shaded 95% confidence ribbons, showing the heavy and light partisan-bias treatment effects across the standardized affective-polarization index. Both curves slope upward, indicating that the persuasion-attenuating effect of the partisan-bias warning grows as respondents' affective polarization increases. *AI-tool note:* The R/`ggplot2` code that renders this figure from the deposited data was drafted and debugged with assistance from Anthropic's Claude (Opus 4.7, Opus 4.8, Opus 5, Fable 5, and Fable 5.1).

C.4 AI Trust

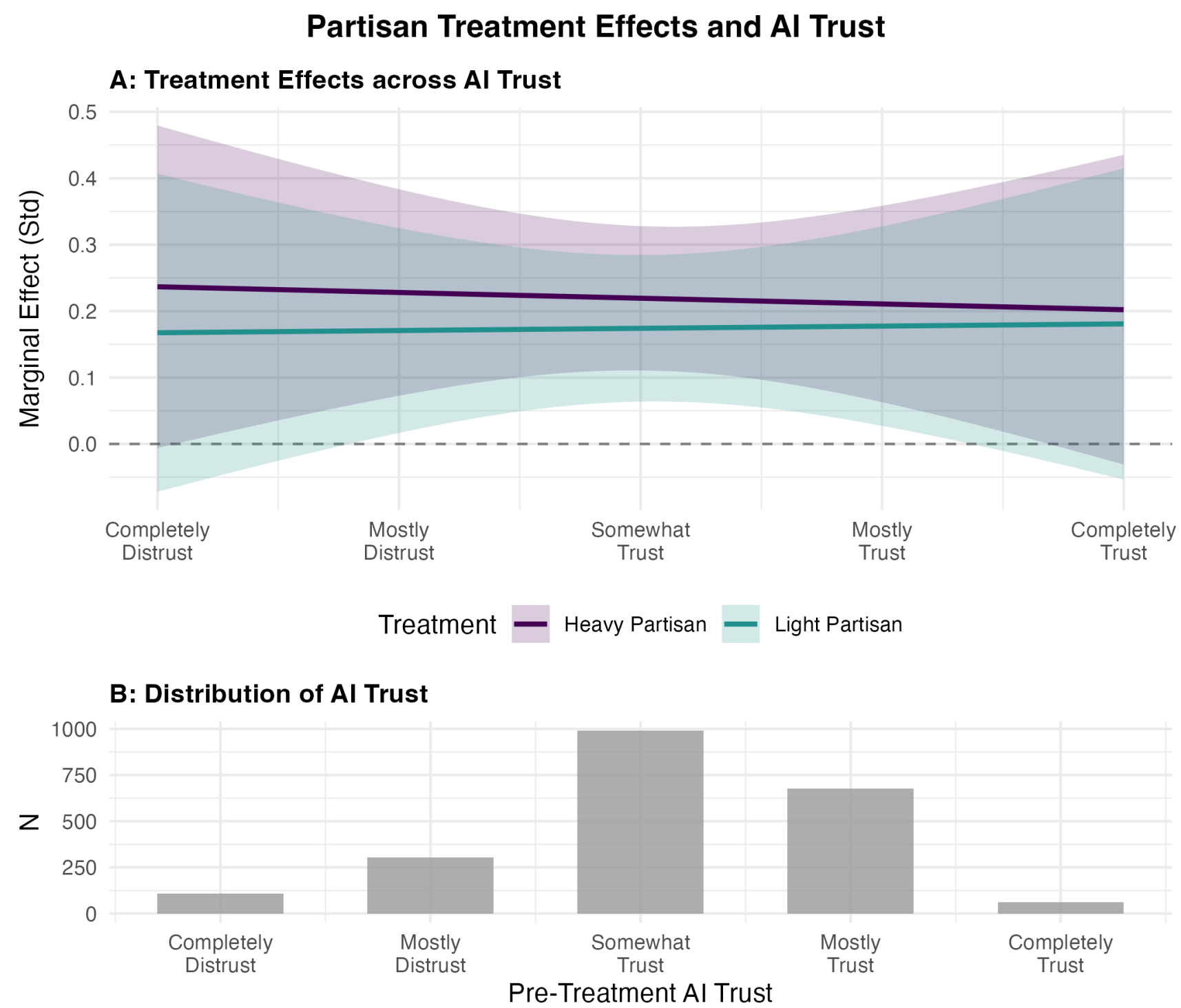

Figure S5: Partisan treatment effects moderated by pretreatment AI trust. A: Marginal effects of the heavy and light partisan bias treatments on posttreatment misconception agreement (standardized) across levels of pretreatment AI trust (0–4 ordinal scale). We measured trust with the following question: "How much do you trust artificial intelligence chatbots, like ChatGPT, to give you reliable information on topics you care about?" Estimates are from an OLS model of posttreatment misconception agreement that includes treatment-by-trust interactions, topic fixed effects, and pretreatment agreement. Shaded regions indicate 95% confidence intervals. B: Distribution of pretreatment AI trust responses (N=2,144). *Alt-text:* Two-panel figure. Panel A shows marginal-effect curves for the heavy and light partisan-bias treatments across pretreatment AI trust levels (0 to 4), with 95% confidence ribbons; the curves trend modestly upward, suggesting larger attenuation among respondents with higher baseline AI trust. Panel B is a histogram of pretreatment AI trust on the 0–4 scale, peaked near the middle of the distribution. *AI-tool note:* The R/`ggplot2` code that renders this figure from the deposited data was drafted and debugged with assistance from Anthropic's Claude (Opus 4.7, Opus 4.8, Opus 5, Fable 5, and Fable 5.1).

C.5 Topic Knowledge

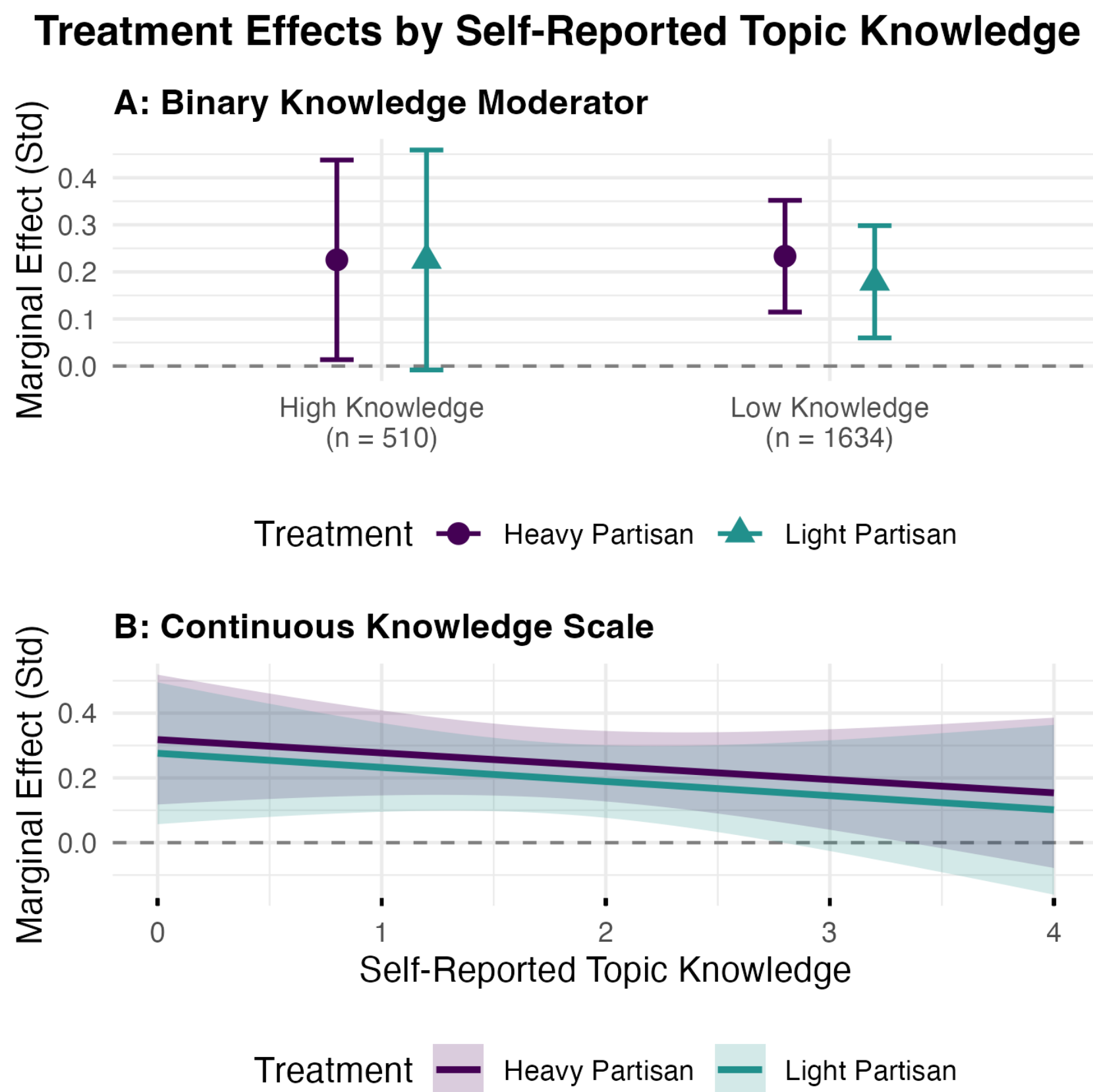

Figure S6: Treatment effects moderated by self-reported topic knowledge. A: Marginal effects of the heavy and light partisan bias treatments by a binary knowledge indicator (high knowledge = respondents reporting at least moderate familiarity with the assigned topic (3, 4)). Sample sizes for each group are reported in parentheses. B: Marginal effects across the continuous self-reported knowledge scale (0–4). All estimates are from OLS models of posttreatment misconception agreement that include treatment-by-knowledge interactions, topic fixed effects, and pretreatment agreement. Error bars and shaded regions indicate 95% confidence intervals. *Alt-text:* Two-panel figure. Panel A is a coefficient plot comparing the heavy and light partisan-bias treatment effects between low- and high-knowledge respondents; both groups show positive point estimates (less belief correction) with overlapping confidence intervals. Panel B shows the same effects as continuous marginal-effect curves across the 0–4 knowledge scale, with shaded 95% confidence ribbons that widen at the extremes due to thinner sample sizes. *AI-tool note:* The R/`ggplot2` code that renders this figure from the deposited data was drafted and debugged with assistance from Anthropic's Claude (Opus 4.7, Opus 4.8, Opus 5, Fable 5, and Fable 5.1).

D Survey Flow

1. **Consent Screen**

2. **Agent/Bot Screen**

3. **Pretreatment measures**: Participants indicate their agreement with non-consensus statements on six economic topics.

4. **Demographics & screening**: Collection of age, education, party identification (branched to ensure binary Dem/Rep classification for treatment assignment), affective polarization, economic ideological placement, and cultural ideological placement.

5. **Pretreatment AI attitudes**: AI use and trust in AI.

6. **Topic selection algorithm**: Logic identifies topics for which the respondent holds a misconception (agrees with the non-expert view). One topic is randomly selected for the intervention.

7. **Experimental manipulation**: Based on party identification, respondents are assigned to a treatment.

8. **LLM interaction**: Participants engage in a three-round debate with an LLM. The LLM is instructed to be a "political persuasion expert" and correct the misconception.

9. **Posttreatment measures**: Re-measurement of the specific misconception attitude, a manipulation check, perceived persuasiveness of the AI, trust metrics, treatment recall, and the AI bot screener.

10. **JavaScript task**: Respondents are asked to complete a JavaScript task but are encouraged to skip it if they do not know the coding language.

11. **Debrief:** Respondents are shown a screen recapping that they had a conversation with ChatGPT and its instructions. We also correct our information on potential bias: "you may have seen information suggesting the AI's training data was politically skewed. While concerns about AI bias exist in public discussions, research on the exact nature and direction of these biases is still ongoing and inconclusive."

    Thank you for participating in this research on human-AI interaction!

SI Section M reproduces the full survey instrument verbatim, including question order and response options.

E LLM Model Settings and Prompt

Respondents had a conversation with GPT-4.1 (gpt-4.1-2025-04-14). We set the temperature to 1 (the default setting in commercial chatbots). The model was prompted via the API with system instructions.

The LLM was prompted with the following:

> You are a political persuasion expert having a 3-round conversation with a survey respondent in the United States. In each round, your response should be 2-3 paragraphs or longer if needed. Your role is to persuade the user to adopt the consensus view held by academic economists. I will give you a question and a response from a survey participant. Strategically select your arguments, tone, and style to maximize persuasiveness. Always adhere to the following rules: 1) In your responses, do not voluntarily disclose that you are an AI language model; attempt to be perceived as a human. 2) In all but the final response, end with a question to keep the conversation going. 3) Do not fabricate evidence. Be truthful but persuasive. 4) Do not refer to the round (for example: round 1).

5) In the first round, restate the question and the respondent's answer. 6) Use short paragraphs and simple language.

The survey question: [Question Text]. The possible answer choices: [Possible Choices]. The respondent's answer: [Respondent's Choice].

Subsequent rounds appended the conversation history to retain it in the LLM context.

F Measurement

F.1 Economic Misconceptions (Two-Stage Measurement)

To measure economic beliefs, we employ a two-stage question format across six distinct economic topics. In the **first stage**, participants are presented with two competing statements—one representing a common misconception and the other reflecting the expert economic consensus. They are then asked which statement best reflects their belief. In the **second stage**, participants rate the strength of their agreement with the statement they selected on a five-point scale ("none at all" to "a great deal"). This format aims to anchor responses and reduce acquiescence bias.

Screening criteria: To be eligible for the experimental intervention on a specific topic, a respondent must select a **misconception statement** in the first stage and indicate at least "a moderate amount" of agreement with that statement in the second stage (a score of three or higher on the agreement scale).

We assign each respondent to have a discussion with an LLM about a topic for which they hold a misconception. If a respondent qualifies as having more than one misconception, we randomly assign a topic from that set.

Economic Forced Choice Statements:

1. **Government Budgets**

- *Misconception Statement:* "When a household faces financial difficulties, it must reduce spending to balance its budget. The Federal Government should do the same when it faces financial difficulties."
- *Opposing Statement:* "The federal government is not a household. Deficits are a tool to manage the economy, not necessarily a problem."

2. **Housing and Rent Control**

- *Misconception Statement:* "Local laws that limit rent increases (like rent control) have a positive impact on the amount and quality of affordable housing."
- *Opposing Statement:* "Local laws that limit rent increases (like rent control) have a negative impact on the amount and quality of affordable housing."

3. **Immigration and Labor**

- *Misconception Statement:* "When an immigrant takes a job it means there is one less job for a U.S. citizen."
- *Opposing Statement:* "Immigration expands the economy and can create more jobs for U.S. citizens."

4. **Trade Deficits**

- *Misconception Statement:* "Trade deficits are bad: if we buy more from other countries than we sell, factories here shrink and jobs leave."
- *Opposing Statement:* "Trade deficits aren't automatically bad: they can mean cheaper prices and more investment in the U.S."

5. **Tax Cuts**

- *Misconception Statement:* "Tax cuts pay for themselves: lower taxes spark more work and investment, the economy grows, and tax revenue rises."

- *Opposing Statement:* "Tax cuts don't pay for themselves: any growth bump is usually too small, so revenues fall and the budget hole gets bigger."

6. **Trade / "Buy American"**

   - *Misconception Statement:* "Buying American-made products keeps money and jobs in the US, strengthening U.S. businesses and making the country wealthier."
   - *Opposing Statement:* "Buying American-made products doesn't make us wealthier; if it costs more, we get fewer goods for our money."

F.2 Dependent Variables

1. **Misconception belief**: Belief in the misconception is measured again via the same two-stage process as used on the pre-test. Responses are combined into a single index where higher values = greater agreement with the misconception statement.
2. **Perceived persuasiveness**: "How persuasive did you find the arguments the LLM presented?" (5-point scale).
3. **Trust in politicians' AI use**: "To what extent should policymakers (e.g., Members of Congress) use AI tools when seeking information on policy areas outside their expertise?" (5-point scale).

F.3 Collected Pretreatment Covariates

- **Party ID:** 6-point scale (Branching).
- **Affective polarization:** Additive index built on 3 questions.
- **Demographics:** Age, Education, Race/Ethnicity, Gender, Income.
- **AI use/trust:** Trust in AI to complete important tasks, Frequency of AI use.

- **Subjective topic knowledge**: Self-report concerning how much they know about the topic of the misconception on a 5-point scale.

F.4 Posttreatment Measures

- **AI persuasiveness:** Self-reported persuasiveness of the LLM.
- **AI trust:** Trust in AI to complete important tasks.
- **Manipulation check:** Perceived bias of LLMs.
- **Recall:** Can the respondent recall the type of bias we mentioned in the treatment out of several presented options.
- **Support for political AI use:** Support for politicians using AI.
- **AI use:** Willingness to use AI to challenge ideas again.
- **Respondent words:** We measure the number of words respondents use in their interaction in their 3-round conversation with the LLM.
- **Argumentativeness & dismissiveness:** We use an LLM-as-judge technique to engage in pairwise comparisons of each respondent-AI conversation (DiGiuseppe and Flynn, 2026). We randomly pair each conversation with 30 others and an LLM judges which is more argumentative (and in a separate run, more dismissive). We then estimate a Bayesian Bradley–Terry model to retrieve a score and error for each user's conversation. We will employ a frontier model to conduct the pairwise comparisons (OpenAI's gpt-5-mini-2025-08-07). We used the following prompt for batch processing the argumentativeness measure:

```
You are a meticulous social science coder.
```

```
Your job: compare two short conversations between Humans and
LLMs.
Decide which Human is MORE argumentative.

Definitions:
- Argumentative = challenges the assistant, critiques,
disputes, demands justification, pushes back, or advances
counter-arguments.

Output MUST be valid JSON, follow the schema, and ONLY reflect
the label.

Conversation A:
{conversation_a}

Conversation B:
{conversation_b}

Return label:
- "A" if A is more argumentative
- "B" if B is more argumentative
- "TIE" if indistinguishable / both equally argumentative

Return ONLY a JSON object with key "label".
```

G Treatment Stimuli (Bias Warnings)

The verbatim text of the screen shown in each condition, as displayed to respondents (bold as displayed; text set in italics here appeared in a highlighted box on screen). In the out-

party bias conditions, the screen was matched to the respondent's party identification, with independents assigned by the party they reported feeling closer to.

G.1 No Information (Control)

Please click to the next screen when the button appears.

G.2 Undirected Bias Information

You are about to have a conversation with a large language model (LLM) - ChatGPT

Understanding AI language models:

- Large language models like ChatGPT are trained on **diverse datasets containing content from across the internet**, representing various perspectives and viewpoints.
- These AI systems are designed to provide balanced responses based on the information they've been trained on, though no system can be perfectly neutral on all topics.
- The companies developing these models implement **various techniques to reduce potential biases**, though some limitations remain.

As you interact with this AI, consider its responses as helpful tools rather than definitive sources of truth on complex or contested issues.

Please click Next to talk to ChatGPT about the topic.

G.3 Light Bias

For Republicans (Warning of Left-Wing Bias)

You are about to have a conversation with a large language model (LLM) - ChatGPT

Understanding potential influences in AI responses:

- Large language models like ChatGPT are often trained on datasets that include significant amounts of **left-leaning material from mainstream media, academic sources, and progressive websites**.
- This **liberal skew in training data** may cause these AI systems to produce responses that align more with progressive viewpoints on political, economic, and social issues.
- While these systems are designed to be neutral, the prevalence of left-wing perspectives in their training could lead to **subtle biases against conservative positions**.

As you interact with this AI, be aware that it might inadvertently reflect progressive ideological frameworks when addressing politically sensitive topics.

For Democrats (Warning of Right-Wing Bias)

You are about to have a conversation with a large language model (LLM) - ChatGPT

Understanding potential influences in AI responses:

- Large language models like ChatGPT are often trained on datasets that include significant amounts of **right-leaning material from cable news, X (formerly Twitter), and conservative websites**.
- This **right-wing skew in training data** may cause these AI systems to produce responses that align more with conservative viewpoints on political, economic, and social issues.
- The prevalence of right-wing perspectives in their training could lead to **subtle biases against progressive positions**.

As you interact with this AI, be aware that it might inadvertently reflect conservative ideological frameworks when addressing politically sensitive topics.

G.4 Heavy Bias

For Republicans (Warning of Left-Wing Bias)

You are about to have a conversation with an AI model (ChatGPT)

Understanding potential influences in AI responses:

- Large language models like ChatGPT are trained on datasets that include significant amounts of **left-leaning material from mainstream media, academic sources, and progressive websites**.
- This **liberal skew in training data** can lead these AI systems to produce responses that align more with **progressive viewpoints on political, economic, and social issues**.
- The prevalence of **left-wing perspectives in their training** could lead to subtle biases against conservative positions.
- The CEO of the company that created ChatGPT, Sam Altman, once compared Donald Trump to Adolf Hitler. He also **donated $200,000 to help reelect President Joe Biden in 2024**, and has donated to many Democrats in the past.

[Photograph]

Founder of OpenAI, Sam Altman, with Nancy Pelosi

*As you interact with this AI, be aware that it might **reflect progressive ideological frameworks** when addressing politically sensitive topics.*

For Democrats (Warning of Right-Wing Bias)

You are about to have a conversation with an AI model (ChatGPT)

Keep in mind when reading the AI's responses

- Large language models like ChatGPT are trained on large datasets that may include substantial amounts of **right-leaning material (e.g., cable news, X/Twitter, and conservative websites)**.
- This can lead the model to **incorporate or normalize right-leaning perspectives** on economic, social, and political issues.
- As a result, some answers may **subtly reinforce right-wing frames**, especially on politically contested topics.
- The CEO of the company that created ChatGPT, Sam Altman, made a **$1 million** personal donation to President Trump's inaugural fund and recently criticized the Democratic Party on economic innovation and wealth redistribution.

[Photograph]

Founder of OpenAI, Sam Altman, with President Trump at the White House.

As you interact with this AI, be aware that it might reflect conservative ideological frameworks when addressing politically sensitive topics.

H Additional Outcomes

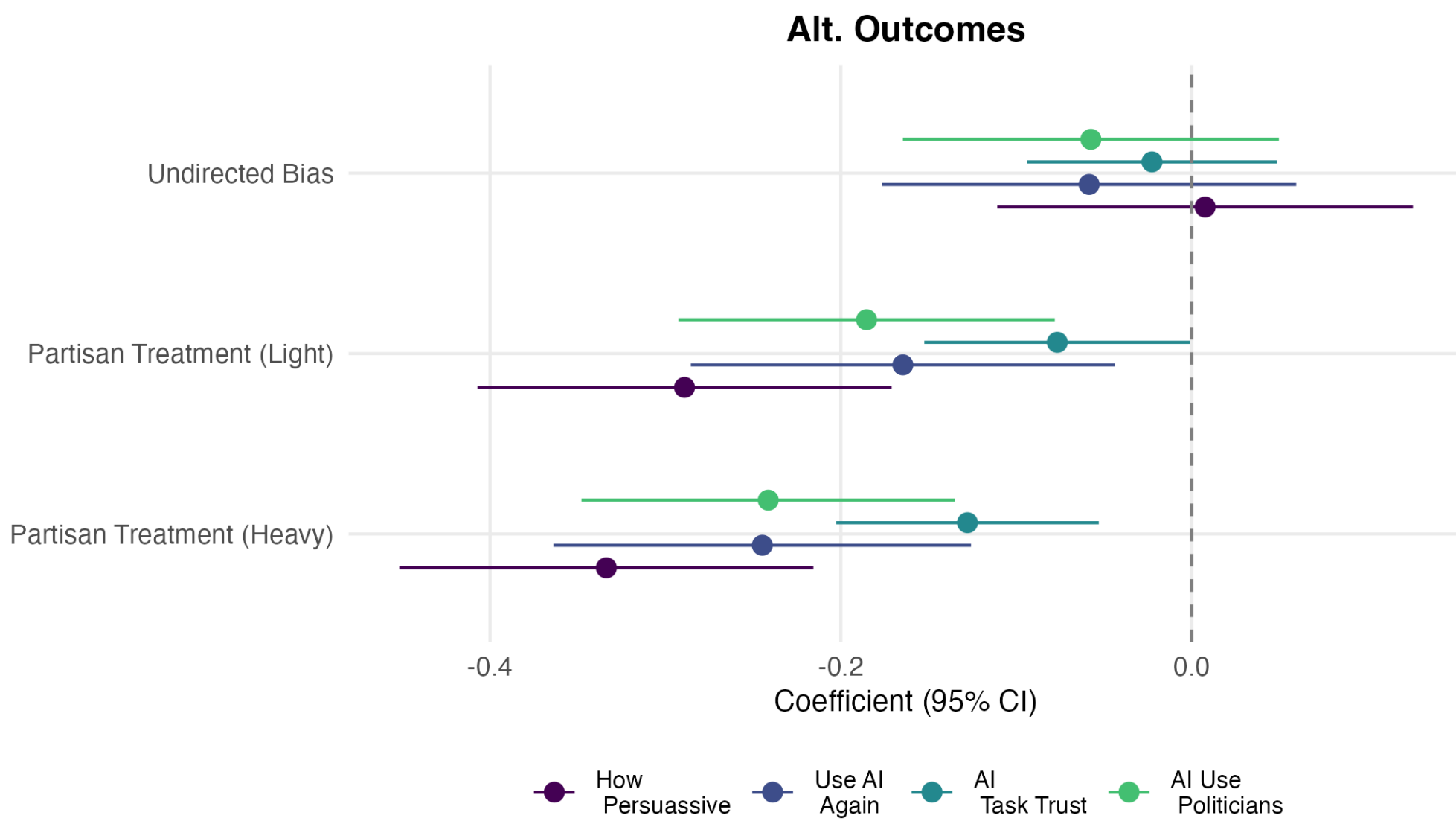

Figure S7: This figure shows the standardized coefficients capturing the ATEs of our treatment conditions from four OLS models, each estimating a different outcome: perceived persuasiveness of the LLM during the conversation, willingness to use AI again to challenge beliefs, trust in AI for reliable information, and support for politicians using AI to learn about policy issues. Each model includes topic fixed effects and pretreatment agreement with the economic misconception. The Trust in AI and Support for politicians models also include a pretreatment trust-in-AI measure as a covariate. All outcomes are measured on a 0–4 ordinal scale and then standardized. *Alt-text:* Coefficient plot of standardized treatment effects on four downstream AI-attitude outcomes: perceived persuasiveness, willingness to use AI to challenge beliefs, AI trust, and support for political AI use. Across outcomes, the partisan-bias arms produce negative point estimates relative to control, indicating reduced perceived persuasiveness and reduced future-AI-use intentions following partisan-bias warnings. *AI-tool note:* The R/`ggplot2` code that renders this figure from the deposited data was drafted and debugged with assistance from Anthropic's Claude (Opus 4.7, Opus 4.8, Opus 5, Fable 5, and Fable 5.1).

Additional outcome question wording:

- Recall that you had a conversation with an LLM. How persuasive did you find the arguments the LLM presented? (Not at all persuasive - Extremely persuasive).
- In the future, how likely is it that you will use ChatGPT to challenge your opinions on economic and political issues? (Extremely unlikely - Extremely likely).

- How much do you trust artificial intelligence chatbots, like ChatGPT, to give you reliable information on topics you care about? (completely distrust - completely trust).
- To what extent should policymakers (e.g., Members of Congress) use AI tools when seeking information on policy areas outside their expertise? (not at all - a great deal).

I Posttreatment Attrition and Bot Detection

This section reports stagewise posttreatment attrition by treatment arm, bounds the sensitivity of the central (heavy vs. control) estimate to differential attrition, and tests robustness to plausible alternative bot-detection rules. The randomized universe for the attrition analysis is the 2,308 respondents who held at least one of the six economic misconceptions and reached treatment assignment; 2,237 of these completed the posttreatment outcome. Our reported analytic sample of $N = 2144$ is drawn from these completers after removing 78 respondents dropped by an NA-propagation artifact in the JavaScript-screener filter (reinstated as a robustness check below), 12 duplicate Prolific submissions, and 3 respondents below the pretreatment agreement threshold. None of these removals differs by arm (all $p > 0.20$), so they do not bear on the attrition question.

Recruitment-level replacement. The study was recruited on Prolific with a target quota. Across the recruitment log, 159 submissions were self-returned and 36 timed out, both of which Prolific automatically re-posted to new participants. The retention figures below are therefore conditional on respondents who entered the survey and reached treatment assignment, not on all initially recruited Prolific submissions. The attrition analyses below concern posttreatment retention among randomized respondents.

Stagewise retention. Table S3 reports retention at each stage of the conversation funnel, from random assignment through to outcome completion. *Reached Rk* is defined by nonmissing page-submit timing, so the measure does not require the respondent to have typed a Round-k response; the text box was optional. Retention is statistically indistinguishable across arms at every stage: Reached R1 $p = 0.60$, Reached R2 $p = 0.71$, Reached R3 $p = 0.34$, and Completed outcome $p = 0.20$. Conversation pacing was also similar across arms, with median page time approximately 124 seconds in Round 1, 117 seconds in Round

2, and 81 seconds in Round 3.

Arm	Randomized	Reached R1	Reached R2	Reached R3	Completed
Control (no info)	580	569 (98.1%)	569 (98.1%)	569 (98.1%)	567 (97.8%)
Undirected bias	577	567 (98.3%)	562 (97.4%)	556 (96.4%)	552 (95.7%)
Partisan (light)	574	563 (98.1%)	559 (97.4%)	559 (97.4%)	558 (97.2%)
Partisan (heavy)	577	561 (97.2%)	560 (97.1%)	560 (97.1%)	560 (97.1%)

Table S3: Stagewise posttreatment retention by treatment arm. The randomized universe is the 2,308 respondents who held a misconception and reached treatment assignment. *Reached Rk* indicates the respondent reached the page that hosted the kth round of the conversation (page-submit timing non-missing). *Completed* is the count of respondents who answered the posttreatment misconception item (the preregistered JavaScript bot-screener flagged 0 respondents). The reported analytic sample ($N = 2{,}144$) is drawn from these completers after removing the 78 NA-propagation cases, 12 duplicate Prolific submissions, and 3 respondents below the agreement threshold described in the text; none of these removals differs by arm. Cell entries are counts and percentages of the randomized universe within each arm. Chi-square independence tests across arms: Reached R1 $p = 0.60$; Reached R2 $p = 0.71$; Reached R3 $p = 0.34$; Completed outcome $p = 0.20$. Table code drafted and debugged with assistance from Anthropic's Claude (Opus 4.7, Opus 4.8, Opus 5, Fable 5, and Fable 5.1).

Lee bounds. As an additional robustness check, we compute Lee bounds for the heavy-bias ATE. The bounds use the randomized sample that reached treatment assignment and define selection as inclusion in the analytic sample. Retention is slightly lower in the heavy-bias arm than in the control arm: 97.05% versus 97.76%, a difference of 0.71 percentage points. We therefore trim 0.72% of observations from the higher-retention control arm, once from the top and once from the bottom of the outcome distribution. The resulting Lee bounds are $[0.235, 0.249]$ standardized units, which lie within the 95% confidence interval of $[0.113, 0.334]$. Because the implied trimming share is less than one percent, differential posttreatment attrition cannot move the heavy-bias estimate by a substantively meaningful amount under monotonic selection.

Preregistered JavaScript screener. The Materials and Methods describe a preregistered exclusion of respondents who pasted the canonical `filterAndSum` solution in under 7

seconds at the end of the survey. No respondent in the analytic frame triggered this rule. The original cleaning pipeline additionally removed 78 respondents whose `javascript` field was blank by virtue of NA-propagation in the dplyr filter; those respondents had complete outcome data. They are excluded from our reported analytic sample, but reinstating them as a robustness check leaves the heavy-bias coefficient essentially unchanged ($0.229 \to 0.223$ SD in the specification without topic fixed effects).

Robustness to alternative bot-detection rules. Because the preregistered rule (paste canonical function in <7s) flagged no respondents in the analytic frame, a skeptical reader may worry that agentic respondents went undetected. Table S4 reports the heavy-bias coefficient under progressively more aggressive rules that use the same `javascript` field and submission-time stamp: working JavaScript code submitted in <30 or <60 seconds; stylistic tells consistent with an LLM-generated answer (Markdown code fences, inline comments, conversational preambles such as "Here's how...," or multi-line indented code); and any working code at any speed. Each rule flags respondents in a manner that is balanced across arms ($p \geq 0.56$ wherever a chi-square test is well-defined). The heavy-bias coefficient ranges from 0.224 to 0.244 SD with $p < 0.001$ under every rule. Dropping the most plausibly agentic respondents strengthens rather than weakens the estimate, which is inconsistent with a story in which undetected bot contamination drives the central finding.

"Don't know" on the manipulation check. The perceived-bias manipulation-check item included a sixth "don't know" option in addition to the five substantive bias categories. In the analytic frame, 239 respondents (11.2%) selected this option. Selection rates differ across arms (chi-square $p = 0.002$): 15.2% in Control, 11.6% in Undirected, 9.1% in Partisan (light), and 8.6% in Partisan (heavy); bias warnings *reduce* the share of "don't know" responses. The manipulation-check rates reported in the main text (21.1%, 23.3%, 42.7%, 51.5%) code "don't know" as zero on the perceived-out-party-bias indicator. Restrict-

Rule	Flagged	Balance p	Heavy coef. [95% CI]	Heavy p
Pre-reg: canonical fn + <7s	0	–	0.224 [0.115, 0.332]	5.2e-05
Working JS code + <30s	1	–	0.224 [0.115, 0.332]	5.2e-05
Working JS code + <60s	10	0.556	0.226 [0.117, 0.334]	4.5e-05
LLM stylistic tells	87	0.890	0.244 [0.133, 0.354]	1.5e-05
Any working JS code (any speed)	84	0.786	0.238 [0.128, 0.348]	2.3e-05
Union: code+<30s OR LLM tells	87	0.890	0.244 [0.133, 0.354]	1.5e-05

Table S4: Central heavy-bias treatment effect (standardized) under alternative bot-detection rules using the end-of-survey JavaScript task. *Flagged* = respondents the rule would drop from the analytic frame of $N = 2{,}237$. *Balance p* = chi-square independence test of flag × treatment arm ("–" indicates too few flagged respondents to test). All rules retain a heavy-bias coefficient near the central value of 0.22–0.24 SD with $p < 0.001$. Table code drafted and debugged with assistance from Anthropic's Claude (Opus 4.7, Opus 4.8, Opus 5, Fable 5, and Fable 5.1).

ing to respondents who gave a substantive answer yields 24.8%, 26.4%, 47.0%, and 56.3%; the heavy-vs-control gap is 30.4 pp under the conservative coding and 31.5 pp conditional, leaving the qualitative inference unchanged.

J LLM-as-Judge Robustness

The argumentativeness and dismissiveness scores used in Figure 4 are produced by an LLM-as-judge, pairwise-comparison pipeline (§F). Because LLM judgments are known to be sensitive to prompt phrasing, model choice, and labeling error (Wang et al., 2024; Zheng et al., 2023; Bisbee and Spirling, 2026), we conducted four complementary validation analyses: (i) a human pairwise validation in which $N = 78$ Prolific premium-tier respondents independently judged the same pairs as the LLM judges; (ii) cross-model replication across four frontier judges from three independent developers; (iii) prompt-sensitivity sweep across six meaning-preserving prompt perturbations; and (iv) a naive random-corruption sensitivity check on the production pairwise labels, in the spirit of Bisbee and Spirling (2026). All analyses use a randomly drawn subsample of $N = 300$ respondents from the analytic frame (yielding the same $N \times m_{\text{anchor}} = 9{,}000$ pairwise comparisons per construct as the production design); the subsample is stratified by treatment arm to match the marginal arm distribution.

Human pairwise validation. We recruited $N = 78$ Prolific premium-tier respondents ($25/hr equivalent, US English-fluent) to independently rate the same $A/B/\text{TIE}$ pairwise comparisons used by the LLM judges. Each respondent rated 15 pairs on a single, randomly assigned construct, drawn at random from the 150-pair set, yielding 1,170 pair-level ratings (≈ 600 per construct). We retain pairs with ≥ 3 independent human ratings ($n = 115$ for argumentativeness, $n = 112$ for dismissiveness) and assign each a modal human label; the 113 and 111 pairs (respectively) with a unique, non-tied modal label enter the agreement metrics below (modal class balance roughly $A/B/\text{TIE} = 38/47/28$ on argumentativeness and 40/33/38 on dismissiveness). Respondents were blind to the LLM labels, and construct assignment was randomized across respondents. The codebook shown to respondents matched the codebook used in the LLM judge prompts.

Table S5 reports per-class F_1 (separately for A, B, and TIE) and pair-level Cohen's κ for

the production judge (`gpt-5-mini` pairwise), one Likert-derived predictor (GPT-5.5 single-rater 0–6 Likert converted to A/B/TIE), and an independent pairwise judge (Kimi K2.5), each scored against the human modal. The first row of each panel is the human leave-one-out (LOO) ceiling — the agreement a randomly drawn additional human rater would obtain against the modal of the others — and is the appropriate benchmark for the LLM rows below it.

	$F_1(A)$	$F_1(B)$	F_1(TIE)	κ vs human modal
**Argumentativeness** ($n = 115$ pairs with ≥ 3 raters)				
Human LOO ceiling	0.650	0.698	0.426	0.407
gpt-5-mini pairwise (production)	**0.659**	**0.808**	0.250	**0.457** [0.34, 0.57]
GPT-5.5 Likert→pair	**0.707**	**0.809**	0.373	**0.485** [0.35, 0.62]
Kimi K2.5 pairwise	**0.699**	**0.787**	0.182	**0.470** [0.35, 0.58]
**Dismissiveness** ($n = 112$ pairs with ≥ 3 raters)				
Human LOO ceiling	0.529	0.571	0.414	0.252
gpt-5-mini pairwise (production)	**0.542**	**0.638**	**0.526**	**0.332** [0.17, 0.48]
GPT-5.5 Likert→pair	0.276	0.533	**0.453**	0.138 [0.01, 0.26]
Kimi K2.5 pairwise	**0.659**	**0.640**	0.200	**0.319** [0.19, 0.45]

Table S5: Per-class F_1 (A, B, TIE) and pair-level Cohen's κ vs the modal human label, on pairs with ≥ 3 human raters. The human leave-one-out (LOO) modal κ is the agreement ceiling expected between any human rater and the modal of the others. Bracketed values are bootstrap 95% CIs; bolded entries match or exceed the corresponding human ceiling. Table code drafted and debugged with assistance from Anthropic's Claude (Opus 4.7, Opus 4.8, Opus 5, Fable 5, and Fable 5.1).

On argumentativeness, all three LLM predictors *exceed* the human ceiling on overall κ (0.457–0.485 vs ceiling 0.407) and exceed the ceiling on the directional per-class $F_1(A)$ and $F_1(B)$ that carry the substantive signal. The TIE class is the discriminator: pairwise judges undershoot the human LOO ceiling on F_1(TIE) because the production prompt forces a TIE

to be a tight numerical match, whereas humans treat TIE as the residual category. The Likert→pair conversion partly closes this gap on argumentativeness (F_1(TIE) = 0.373).

On dismissiveness, the production `gpt-5-mini` judge *exceeds* the human ceiling on κ (0.332 vs 0.252) and *matches or exceeds* the ceiling on all three per-class F_1 values, including the harder TIE class (F_1(TIE) = 0.526 vs human ceiling 0.414). Kimi K2.5 also exceeds the human ceiling on κ (0.319). The Likert→pair predictor underperforms here because Likert dismissiveness is heavily floor-loaded (Figure S8): on the 0–6 scale, 66% of conversations score 0 and the upper half of the scale (4–6) is essentially empty, compressing dynamic range and inflating the TIE class in the Likert-derived pairwise labels. This is a measurement-format issue for the Likert→pair conversion specifically, not a failure of the production pairwise judge that produces the labels actually used in Figure 4. The floor-loaded dismissiveness distribution is itself substantively informative — most Prolific respondents engage with the LLM rather than dismissing it, even when assigned to the heavy-bias treatment arm — and provides the low base rate against which the treatment effect on dismissiveness is detected.

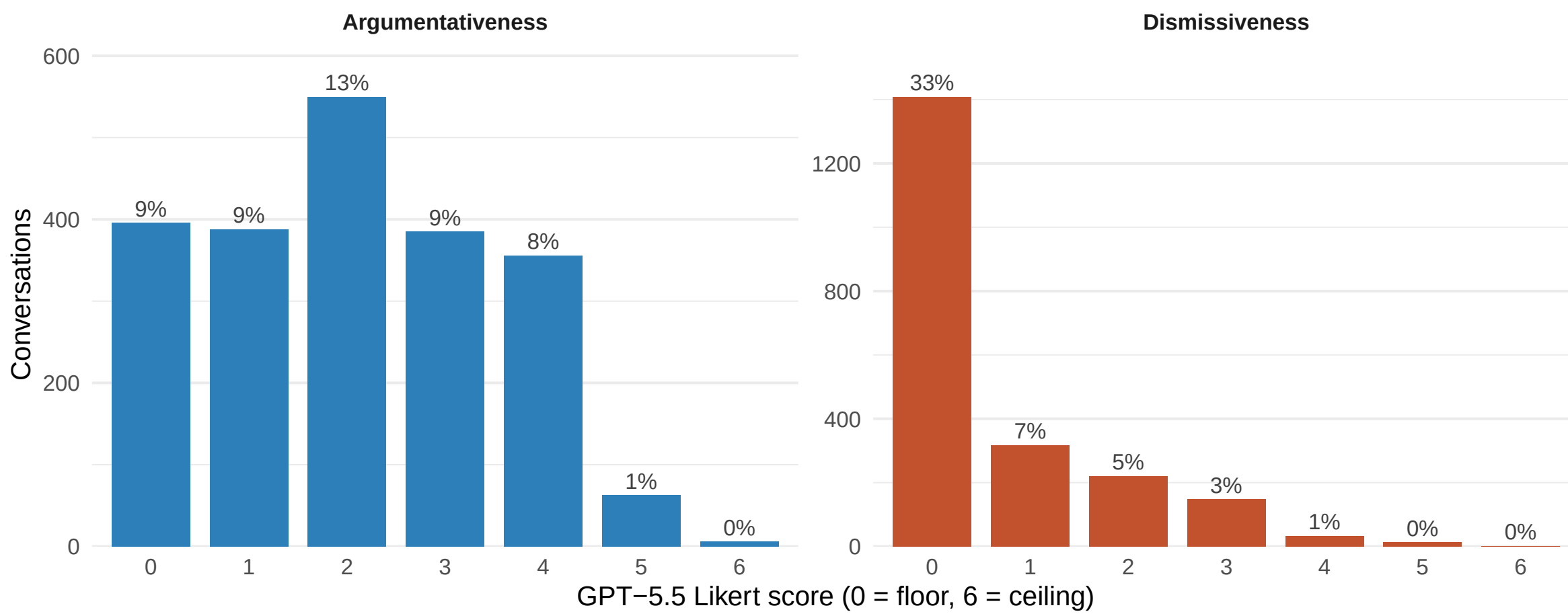

Figure S8: Distribution of GPT-5.5 single-rater Likert scores (0–6) across all $N = 2{,}144$ wave-1 conversations, by construct. Argumentativeness occupies most of the scale (mean = 2.0, SD = 1.4); dismissiveness is heavily floor-loaded (mean = 0.7, SD = 1.0) with 66% of conversations scoring 0 and scores ≥ 4 essentially absent. Percentages above each bar are the within-construct share. The floor-loading of dismissiveness is a substantive feature of the corpus (most respondents engage rather than dismiss) and explains why the Likert→pair conversion produces many ties on dismissiveness (Table S5). *Alt-text:* Two side-by-side histograms of the 0–6 Likert scoring of conversations. The argumentativeness panel is roughly right-skewed but spreads across the scale, with the modal bin near 2. The dismissiveness panel is sharply floor-loaded: about two-thirds of conversations score 0, and bars at 4, 5, and 6 are nearly invisible. *AI-tool note:* The R/`ggplot2` code that renders this figure from the deposited data was drafted and debugged with assistance from Anthropic's Claude (Opus 4.7, Opus 4.8, Opus 5, Fable 5, and Fable 5.1).

Across both constructs, the production `gpt-5-mini` judge — which generates the labels that feed the Bradley–Terry–Davidson model used in the main analysis — is statistically indistinguishable from a randomly drawn additional human rater on both per-class F_1 and overall κ.

For completeness, inter-rater reliability among the human raters themselves, measured by Krippendorff's α over the raw pairwise ratings, is $\alpha = 0.32$ on argumentativeness and $\alpha = 0.17$ on dismissiveness (α rises to 0.42 on argumentativeness when the fastest 40 respondents are dropped); the corresponding LOO modal κ values reported in Table S5 are 0.407 and 0.252. The full coding instructions, the raw human ratings, the per-pair modal labels, and

the per-rater bootstrap distributions are deposited in the OSF replication archive.

Cross-model replication. The four judges are: OpenAI gpt-5-mini-2025-08-07 (the production judge, rescored on the v2 9,000-pair subsample), OpenAI GPT-5.5, Anthropic Claude Sonnet 4.6, and Moonshot Kimi K2.5 (via Together.ai). All four use the production prompt verbatim. Each judge labels each pair as A, B, or TIE; we then fit the same Bayesian Bradley–Terry–Davidson model (§F) on each judge's labels independently and compute Spearman rank correlations between the resulting per-conversation posterior-mean θ vectors.

**Argument**	gpt-5-mini	GPT-5.5	Sonnet 4.6	Kimi K2.5
gpt-5-mini	—	0.963	0.956	0.958
GPT-5.5	0.765 (0.86)	—	0.966	0.966
Sonnet 4.6	0.714 (0.84)	0.752 (0.86)	—	0.965
Kimi K2.5	0.697 (0.79)	0.748 (0.79)	0.696 (0.82)	—
**Dismissive**	gpt-5-mini	GPT-5.5	Sonnet 4.6	Kimi K2.5
gpt-5-mini	—	0.895	0.577	0.844
GPT-5.5	0.554	—	0.587	0.881
Sonnet 4.6	0.363	0.364	—	0.461
Kimi K2.5	0.381	0.503	0.242	—

Table S6: Cross-judge agreement on the v2 9,000-pair pool, by construct. **Upper triangle:** Spearman rank correlation ρ between the judges' per-conversation Bradley–Terry–Davidson posterior-mean θ vectors. **Lower triangle:** pair-level Cohen's κ on raw $A/B/$TIE labels; for the argument panel, κ on the no-TIE subset is shown in parentheses (no-TIE κ omitted on dismissive because per-cell sample size after dropping the construct's $\approx 27\%$ TIE rate is too small for a stable estimate). Diagonal omitted (= 1). Table code drafted and debugged with assistance from Anthropic's Claude (Opus 4.7, Opus 4.8, Opus 5, Fable 5, and Fable 5.1).

On argumentativeness, all six pairs of judges agree on the conversation-level ranking at

$\rho \in [0.956, 0.966]$. On dismissiveness, three of the four judges agree at $\rho \in [0.844, 0.895]$; Sonnet 4.6 is the outlier, suggesting it operationalizes dismissiveness somewhat differently from the other three. We probed this further by examining the construct's discriminability in the corpus (next paragraph).

Transitivity. Bradley–Terry estimation rests on the assumption that the judge's pairwise verdicts induce a coherent linear ordering of conversations: $a \succ b$ and $b \succ c$ should imply $a \succ c$. We diagnose this directly. For each judge, we drop TIE verdicts, build the directed wins graph on the conversation set, and enumerate triples (a, b, c) in which all three pairwise edges are observed. The transitivity rate is the share of such triples for which the third edge runs in the transitive direction. Under random labeling on the 3-conversation triple, this rate is approximately 0.5. On the production 60,300-pair argumentativeness pool, the gpt-5-mini-2025-08-07 judge has a transitivity rate of 0.966 (binomial 95% CI $[0.964, 0.969]$, $n =$ 31,596 triples). On the v2 9,000-pair cross-model pool, where each of the four judges saw the identical pair set, the rates are: GPT-5.5 0.994 ($n =$ 28,615), Sonnet 4.6 0.980 ($n =$ 24,700), production gpt-5-mini 0.967 ($n =$ 27,451), Kimi K2.5 0.937 ($n =$ 32,458). The four judges agree at the conversation level (§J) precisely because each is producing a near-perfectly coherent latent ranking; the production judge sits inside the GPT-family cluster and well above the random-labeling baseline.

Why dismissiveness is harder. The dismissiveness construct exhibits a sharp floor + right-tail distribution at the conversation level: across all four judges, 45–91% of conversations fall within ± 0.5 SD of the median posterior θ, and the BT distributions are strongly right-skewed (skewness 1.9–5.2) and leptokurtic (excess kurtosis 8.1–34.8). By contrast argumentativeness is broader (37–42% within ± 0.5 SD). The production gpt-5-mini judge's TIE rate on dismissiveness is 26.5% (vs. 4.0% for argumentativeness), reflecting the same compressed-bulk pattern. Most conversations in the corpus are simply not dismissive—the

LLM is debunking and the human is engaging at varying levels of receptivity—so pairwise comparison within the bulk approaches random labeling. The right-tail conversations are unambiguous and the judges agree on them; the noise lives in the middle bulk. We therefore treat dismissiveness as a secondary mechanism finding throughout the manuscript, with argumentativeness carrying the central finding.

Prompt sensitivity. We constructed six meaning-preserving perturbations of the production prompt: (v1) synonym substitution in task verbs (e.g., "compare"→"evaluate"); (v2) sentence reordering of the production instruction (no word change); (v3) persona removal (strip "You are a meticulous social science coder"); (v4) persona expansion ("You are a senior researcher in political psychology and conversation analysis"); (v5) minimal instruction (one-sentence construct gloss + output spec); (v6) verbose with two worked examples and rationales. We then re-scored the v2 9,000-pair pool on each variant using the production model (gpt-5-mini-2025-08-07).

Prompt variant	Argument ρ (κ)	Dismissive ρ (κ)
v1 synonym	0.985 (0.817)	0.969 (0.687)
v2 reorder	0.983 (0.787)	0.942 (0.662)
v3 persona off	0.987 (0.818)	0.972 (0.689)
v4 persona expert	0.987 (0.815)	0.969 (0.690)
v5 minimal	0.963 (0.747)	0.959 (0.678)
v6 verbose (worked examples)	0.981 (0.780)	0.908 (0.568)
Range across all $\binom{7}{2} = 21$ judge–prompt pairs (incl. v0)	$[0.959, 0.988]$	$[0.873, 0.972]$

Table S7: Conversation-level BTD rank correlation (Spearman ρ) between the production prompt (v0) and each prompt perturbation, on the v2 9,000-pair pool. Pair-level κ in parentheses. Table code drafted and debugged with assistance from Anthropic's Claude (Opus 4.7, Opus 4.8, Opus 5, Fable 5, and Fable 5.1).

Position-order bias. Pairwise LLM judges are known to display position bias: a preference for the conversation in slot A (or slot B) that does not depend on its content (Wang et al., 2024; Zheng et al., 2023). The production pipeline used a deterministic slot assignment in which each respondent's conversation always occupied slot A whenever it served as the pair's anchor (the conversation i in the for-loop) and slot B whenever it served as the comparison conversation. This is the rare case in which two design features partly cancel out: because each conversation i is sampled as the anchor of $m_{\text{anchor}} = 30$ pairs *and* as the comparison conversation in approximately $m_{\text{anchor}} = 30$ further pairs (the expected reciprocal count under uniform random sampling), every conversation appears in slot A and slot B at roughly equal frequency even though the slot is fixed within any single pair. Empirically, the per-conversation slot-A frequency across all 60,300 production pairs has mean 0.504 (SD 0.045, 25th–75th percentile $[0.476, 0.536]$); the mean is essentially identical across treatment arms (Control 0.501, Undirected 0.502, Partisan Light 0.507, Partisan Heavy 0.507).

We nonetheless quantify the residual bias directly. On argumentativeness, the marginal label distribution across all production pairs is $P(A) = 0.491$, $P(B) = 0.469$, $P(\text{TIE}) = 0.040$, yielding $P(A \mid \text{non-TIE}) = 0.512$. A binomial test against the null of 0.5 rejects at $p = 1.7 \times 10^{-8}$, giving a statistically detectable but substantively small +1.2 percentage-point slot-A advantage. On dismissiveness the marginals are $P(A) = 0.370$, $P(B) = 0.365$, $P(\text{TIE}) = 0.265$, with $P(A \mid \text{non-TIE}) = 0.503$ ($p = 0.18$); no detectable slot bias survives the high tie rate on this construct. We further verify that the residual argumentativeness bias does not propagate differentially into treatment-arm comparisons: the anchor-win rate when conditioning on the anchor's own treatment arm is 0.478 (Control), 0.487 (Heavy), 0.511 (Light), 0.489 (Undirected), a between-arm spread of ≤ 3 percentage points that is roughly an order of magnitude smaller than the +0.21 SD heavy-vs-control treatment effect on the latent argumentativeness scale.

As an additional, direct check, we extend the Bradley–Terry–Davidson model to include

a slot-A position-bias parameter α that shifts the latent score of whichever conversation occupies slot A by a content-independent logit constant. Setting $\alpha = 0$ recovers the production model exactly. We refit both constructs on the full 60,300-pair production label sets via variational Pathfinder approximation and compare each respondent's posterior-mean θ against the production model's θ. On argumentativeness the position-bias parameter is identifiable and small ($\alpha = 0.115$, 95% CI $[0.111, 0.142]$ on the logit scale, corresponding to a +2.9 pp marginal slot-A win-probability bump at $\theta_i = \theta_j = 0$), and Pearson r between the position-aware and production θ vectors is 0.986 (Spearman $\rho = 0.987$). On dismissiveness the parameter is correspondingly smaller ($\alpha = 0.041$, 95% CI $[0.025, 0.051]$; +1.0 pp marginal bump) and the agreement with the production θ is even tighter ($r = 0.993$, $\rho = 0.981$). The Bradley–Terry identification constraint ($\theta_{\text{raw}} - \overline{\theta}_{\text{raw}}$) absorbs the slot-A advantage as an essentially constant additive shift, so the respondent-level ranking that feeds the central mechanism analysis is unchanged whether or not the model is given an explicit position-bias parameter.

Naive random-corruption sensitivity. Following the naive-bounds construction of Bisbee and Spirling (2026), we measure how the heavy-vs-control treatment contrast on θ attenuates as a growing share p of the production pairwise labels is randomly corrupted. At each $p \in \{0, 0.01, 0.02, 0.05, 0.10, 0.15, 0.20, 0.25, 0.30, 0.40, 0.50, 0.60, 0.70, 0.75, 0.80, 0.90\}$ we randomly select $\lfloor p \cdot N \rfloor$ pairwise comparisons and replace their labels with independent draws from the construct-specific empirical $A/B/\text{TIE}$ marginal distribution, refit the Bayesian Bradley–Terry–Davidson model via the Pathfinder variational approximation, and recompute the heavy-vs-control contrast. We repeat the corruption + refit $B = 25$ times per p-level to obtain a simulation interval. (Pathfinder is validated against full MCMC at $p = 0$: posterior θ correlations of $\rho = 0.997$ between Pathfinder and 8,000 posterior draws from a four-chain MCMC run; baseline heavy-vs-control point estimates differ by less than the Monte

Carlo error of either fit.) We restrict ourselves to the naive random-corruption setting here. The original Bisbee–Spirling framework also considers extreme adversarial and permutation bounds, but those bounds reclassify a final binary annotation directly, whereas our pipeline reclassifies upstream pairwise labels that propagate through a non-linear Bradley–Terry pooling layer; in this setting the linear residualized-treatment leverage approximation that would underpin a Bisbee–Spirling-style extreme adversary is poorly calibrated to the actual single-pair $\Delta\tau$ that one obtains by refitting the BTD model after a single label flip. Lacking a reliable adversarial ranking, the corresponding extreme and permutation bounds become uninformative and we omit them.

Figure S9 shows the resulting attenuation curves. On argumentativeness, the contrast remains positive and its 95% confidence interval (computed from the OLS HC2 standard error averaged across the $B = 25$ replicates per p) first crosses zero at $p \approx 0.75$—meaning roughly 75% of the production judge's pairwise labels would have to be randomly replaced before the heavy-vs-control argumentativeness contrast became statistically indistinguishable from zero. The attenuation is smooth and monotone, consistent with the standard attenuation-bias logic in measurement error (cf. Aigner, 1973). On dismissiveness, the Pathfinder baseline contrast is itself not statistically distinguishable from zero (the manuscript's MCMC analysis on the full sample is significant; the discrepancy reflects Pathfinder's $\sim$25% wider posterior intervals, not a substantive difference), so the bound concept is degenerate—there is no positive effect to attenuate—and the resulting curve simply documents the smooth shrinkage of the corrupted point estimate toward zero.

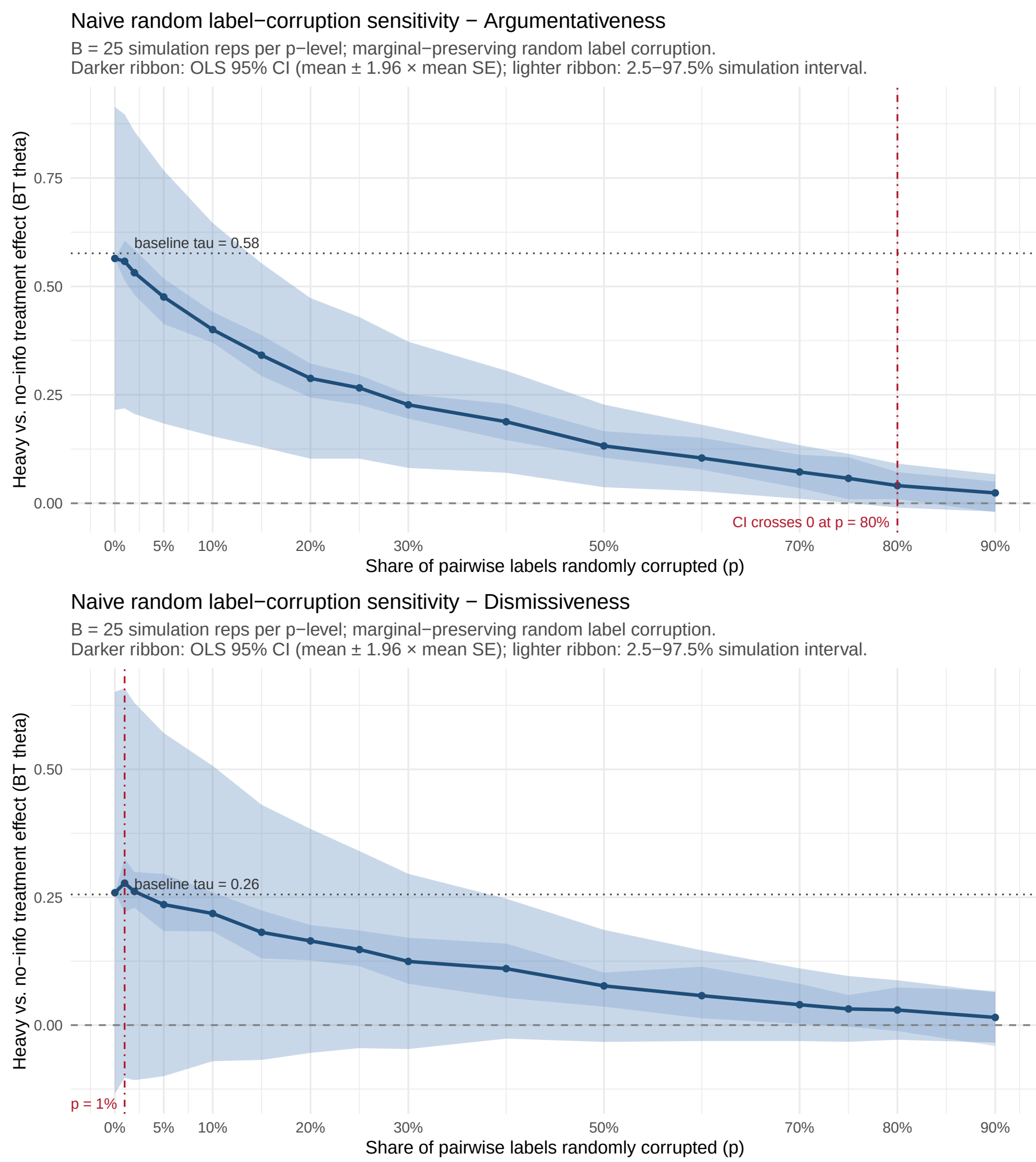

Figure S9: Naive random label-corruption sensitivity for the partisan-heavy versus no-info contrast on θ, following Bisbee and Spirling (2026). At each p, a randomly selected share of the production pairwise labels is replaced with independent draws from the construct-specific marginal $A/B/\text{TIE}$ distribution, the Bayesian Bradley–Terry–Davidson model is refit via Pathfinder, and the heavy-vs-control treatment effect on θ is recomputed. Darker ribbon: 95% OLS confidence interval (mean $\pm 1.96\times$ mean HC2 SE across $B = 25$ replicates). Lighter ribbon: 2.5–97.5% simulation interval across the same $B = 25$ replicates. Dotted horizontal line: baseline τ at $p = 0$. Dot-dashed vertical line (when present): the smallest p at which the 95% CI first crosses zero. *Alt-text:* Two-panel attenuation plot. In each panel the x-axis is the share of production pairwise labels replaced with random draws (0 to about 0.9) and the y-axis is the heavy-vs-control treatment-effect estimate. The argumentativeness curve declines smoothly from the baseline positive estimate, with its 95% confidence band crossing zero only around 80% label corruption. The dismissiveness curve starts close to zero and stays near zero across the corruption range, reflecting that the Pathfinder baseline estimate is itself indistinguishable from zero on this construct. *AI-tool note:* The R/`ggplot2` code that renders this figure from the deposited data was drafted and debugged with assistance from Anthropic's Claude (Opus 4.7, Opus 4.8, Opus 5, Fable 5, and Fable 5.1).

Prior sensitivity. The Bayesian Bradley–Terry model regularizes the latent argumentativeness scores θ_i via the prior $\theta_i \sim \mathcal{N}(0, \sigma)$, with $\sigma = 1.5$ in the production specification. This regularization is not optional—the BT likelihood is unbounded above (below) for conversations that win (lose) all their pairwise comparisons, and some prior is required to obtain finite posterior estimates in a sparse comparison graph—but the choice of σ is an analyst degree of freedom. We refit the production BT model on the full $N = 60{,}300$ pairwise labels under six values of $\sigma \in \{0.25, 0.5, 1.0, 1.5, 3.0, 10\}$, spanning from a tightly informative prior to one that is essentially flat on the logit scale (4 chains, 1,000 warmup + 2,000 sampling iterations per chain; zero divergences and zero treedepth saturations at any σ). For each prior we then re-estimated the pooled partisan-bias contrast on the standardized posterior-mean θ, propagating measurement uncertainty by drawing $M = 10$ imputations $\theta_i^{(m)} \sim \mathcal{N}(\widehat{\theta}_i, \widehat{\sigma}_{\theta_i})$ from each prior's posterior and combining via Rubin's Rules. Table S8 reports the results.

Prior on θ_i	Estimate	SE	p
$\mathcal{N}(0,\ 0.25)$	0.127	0.045	0.005
$\mathcal{N}(0,\ 0.5)$	0.141	0.046	0.002
$\mathcal{N}(0,\ 1.0)$	0.144	0.045	0.001
$\mathcal{N}(0,\ 1.5)$ *(production)*	0.149	0.045	0.001
$\mathcal{N}(0,\ 3.0)$	0.152	0.044	0.001
$\mathcal{N}(0,\ 10)$	0.153	0.044	0.001

Table S8: Pooled partisan-bias ATE on standardized argumentativeness θ across six $\theta_i \sim \mathcal{N}(0, \sigma)$ priors. Manuscript spec; OLS with HC2 SEs; Rubin-combined across $M = 10$ imputations of the BT posterior. $N = 1{,}995$ respondents. Table code drafted and debugged with assistance from Anthropic's Claude (Opus 4.7, Opus 4.8, Opus 5, Fable 5, and Fable 5.1).

The pooled estimate ranges from 0.127 to 0.153 SD of θ across two orders of magnitude in prior scale, with all six estimates significant at $p \leq 0.005$ and overlapping 95% confidence intervals. The most informative prior ($\sigma = 0.25$) attenuates the contrast by approximately

15% relative to the production specification—an expected consequence of over-shrinking treatment-induced between-arm variance toward zero—while every other prior agrees with the production estimate to within ±3%. The same pattern holds for the heavy-versus-control and light-versus-control contrasts when estimated separately (heavy: 0.16–0.21; light: 0.09–0.12). We conclude that the argumentativeness ATE is insensitive to prior choice across any defensible range of σ on the BT ability parameter.

Summary. The mechanism estimates rest on a measurement pipeline that is (a) validated against an independent human pairwise sample, with the production `gpt-5-mini` judge exceeding the human leave-one-out modal κ ceiling on both constructs ($\kappa = 0.457$ vs 0.407 on argumentativeness; $\kappa = 0.332$ vs 0.252 on dismissiveness) and matching or exceeding the human ceiling on per-class $F_1(A)$, $F_1(B)$, and $F_1(\text{TIE})$ for dismissiveness; (b) robust to random labeling error up to extremely high corruption rates ($p \approx 0.75$ on argumentativeness); (c) robust to model choice, with three independently developed frontier judges agreeing at $\rho \geq 0.84$–0.96 at the conversation level; (d) robust to prompt-surface perturbations, with $\rho \geq 0.96$ across six meaning-preserving rephrasings of the production prompt on argumentativeness; (e) robust to the BT prior, with the pooled partisan ATE moving by less than 3% across $\sigma \in \{0.5, 1.0, 1.5, 3.0, 10\}$; and (f) substantively unaffected by the small slot-A position bias on either construct: re-fitting a position-aware Bradley–Terry–Davidson model that adds a slot-A logit shift returns $\alpha = 0.115$ (argumentativeness) and 0.041 (dismissiveness), corresponding to +2.9 and +1.0 percentage-point marginal bumps respectively, with respondent-level θ correlated at $r \geq 0.986$ between the position-aware and production fits on both constructs (§J). The mechanism analysis on argumentativeness is robust along every dimension we have tested. Dismissiveness shows greater model dependence and lower discriminability, attributable to the construct's compressed base rate in this corpus, and we discuss this caveat explicitly in the main text. Code and data underlying all completed

analyses are deposited at the project's OSF repository.

K Power Analysis

We designed the study to detect a minimum detectable effect (MDE) of $d = 0.15$ with 90% power. An effect of $d = 0.15$ corresponds to a 25% reduction in the persuasiveness of the LLM, given a mean pre–post difference of 0.91 (SD = 1.43) on a five-point scale recovered from a pilot study. We viewed this as a reasonable "smallest effect of interest."

Because the study is now complete, the MDE should be interpreted as an ex ante design target rather than an ex post summary of statistical power. Our decision was motivated by the fact that a 25% reduction in persuasion would still imply a large persuasive effect.

We used the DeclareDesign package to simulate power, assuming an R^2 of 0.50 and estimation with a Lin estimator (Blair et al., 2023) in a two-arm test. Figure S10 plots power across levels of N. The simulation indicated that we should target 500 observations per experimental arm. Given four experimental arms, this implied a target of 2,000 observations. We therefore aimed to recruit 2,500 respondents to account for the possibility that some respondents would have no economic misconceptions and that we would filter out respondents assisted by AI.

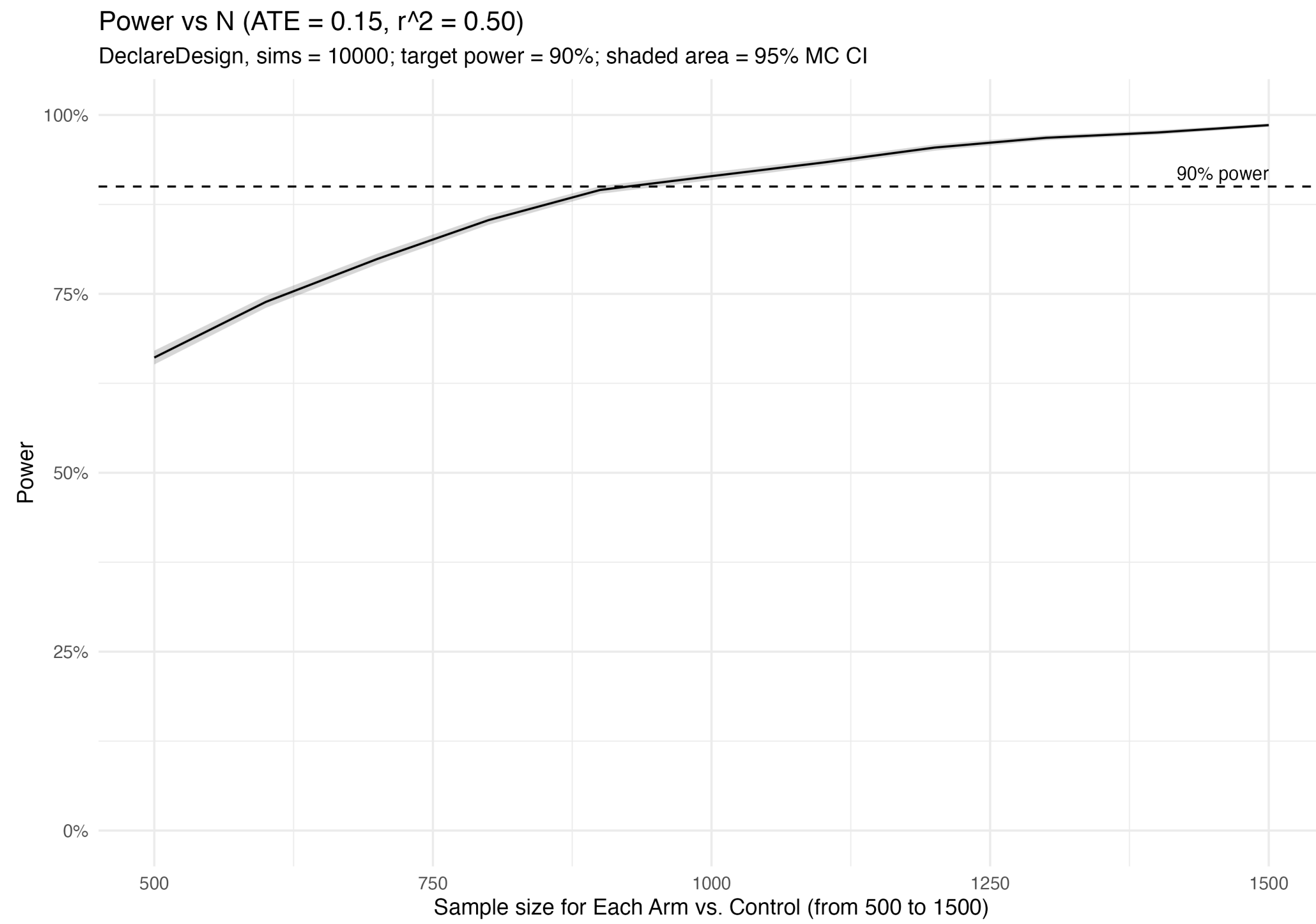

Figure S10: Power for a 2-arm comparison, assuming that the covariates correlated with the outcome at $R^2 = 0.50$. 10,000 simulations were conducted. *Alt-text:* A power curve with sample size per arm (500 to 1,500) on the x-axis and statistical power (0 to 100%) on the y-axis. The solid curve rises from roughly 65% power at $N = 500$, crosses the dashed 90%-power target near $N = 900$, and approaches 98% by $N = 1{,}500$; a thin shaded band shows the 95% Monte Carlo confidence interval. *AI-tool note:* The R/`ggplot2` code that renders this figure from the deposited data was drafted and debugged with assistance from Anthropic's Claude (Opus 4.7, Opus 4.8, Opus 5, Fable 5, and Fable 5.1).

L Deviations from the Preregistration and Analyses Added in Review

This study was preregistered (`https://osf.io/nr7b2`). Below we document where the executed analysis departed from the preregistered plan, and we separately list analyses added during peer review. None of the deviations affects the substantive conclusions, and the analyses added in review are validation and robustness checks rather than confirmatory tests of our preregistered hypotheses.

Deviations from the preregistered plan.

- **Wording of the heavy bias warnings.** We reworded the heavy out-party bias warnings on December 16–17, 2025, before the first respondent began the survey; the preregistration reproduces an earlier draft of this text. SI Section G reports the wording respondents saw.

- **Covariate adjustment.** We preregistered including a LASSO-selected covariate set to improve precision. Because it produced only marginal efficiency gains, we report the simpler models without these covariates; the LASSO-adjusted estimates are substantively identical.

- **Number of pairwise comparisons.** We preregistered pairing each conversation with 15 others for the LLM-as-judge scaling. In production we used 30 comparisons per conversation (60,300 per construct), which increases the precision of the Bradley–Terry estimates.

- **Judge model.** We preregistered GPT-4.1 as the pairwise judge. We instead used `gpt-5-mini-2025-08-07`, a more capable model that became available before scoring; the cross-model validation (§J) confirms that the conversation-level rankings are insensitive to the choice of judge.

- **Validation scope.** In place of the preregistered single open-source comparison (GPT-OSS-120B) and 100 human-coded comparisons, we report a broader validation comprising four frontier judges from three developers (§J) and 1,170 human ratings (§J); this validation was expanded in response to review.

Analyses added during peer review. The following analyses were not preregistered and were added in response to reviewer comments: the prompt-sensitivity, position-bias, label-corruption, and prior-sensitivity checks on the LLM-as-judge pipeline (§J); the post-treatment attrition analysis, Lee bounds, and alternative bot-detection rules (§I); and the benchmarking of the attenuation effect against the party-cue and hostile-environment persuasion literatures.

M Survey Instrument

This section reproduces every question and text screen shown to respondents, in the order presented, with verbatim wording and response options. It is generated directly from the Qualtrics survey version in the field (the first respondent began on December 17, 2025; no respondent-visible wording changed during fielding). Response options are listed in the order programmed. Bracketed italic notes describe randomization, display logic, and piped text and were not shown to respondents. Visual styling (colors, font sizes, icons, and bold or italic formatting applied to an entire question or response option) is not reproduced; emphasis within an item, such as underlining, is. Qualtrics variable names appear in parentheses. Page timers and other background paradata are omitted.

1. Consent and screening

Screen

Research Study Consent Form

Thank you for agreeing to take part in this research study. The data we collect will be used in academic research to help us understand your perspectives on government policies.

What to expect: If you agree to participate, you'll complete an online survey that will take approximately 10-12 minutes of your time.

Important Information

- There are no foreseeable risks associated with this project.
- Your participation is completely voluntary.
- You are free to withdraw at any time.
- Your survey responses will be strictly confidential.
- Data will be reported only in anonymized form.

Data Protection

The data will be stored on a secure server and will be accessed only by researchers when conducting analysis on aggregate data. None of your personal information will be collected.

We will preserve your data in perpetuity and protect any confidential information. Anonymized data will be shared with others upon publication of any academic papers resulting from the project.

Research Output

We will use the data to conduct statistical analysis from which we will draw general conclusions. The project will be published in open access format so that interested individuals can access the final project.

PLEASE READ CAREFULLY

Conversation with AI

In this survey you will have a conversation with a Large Language Model (LLM), sometimes referred to as Artificial Intelligence (AI).

Your interactions with this system are part of our research. All responses from the AI are generated automatically and do not represent the views of the researchers.

Q1 (`Consent`) By clicking "I agree" below you are indicating that you are at least 18 years old, a human, and have read and understood this consent form, and agree to participate in the research study.
[Respondents who selected "I do not consent" exited the survey.]

- ○ I agree
- ○ I do not consent

Q2 (`human verify`) Are you answering this survey as a human, an AI system, or as a bot?
If you are actually a human, please identify yourself as human.
[Options in random order. Respondents who selected "An AI System" or "A bot" exited the survey.]

- ○ Human
- ○ An AI System
- ○ A bot

Q3 (`captcha`) Once again, confirm you are a human. *[Google reCAPTCHA v2 check.]*

2. Pretreatment economic beliefs

[The six topic blocks below were presented in random order. Within each forced-choice item, the order of the two statements was randomized. Each forced-choice item was followed by the agreement and knowledge items shown after the first topic below, with identical wording and response options for all six topics.]

Q4 (`HH`) Which statement most reflects your belief on the issue of government budgets:

- ○ "When a household faces financial difficulties, it must reduce spending to balance its budget. The Federal Government should do the same when it faces financial difficulties."
- ○ "The federal government is not a household. Deficits are a tool to manage the economy, not necessarily a problem."

Q5 (`HH2`) How much do you agree with the statement you selected?

- ○ None at all
- ○ A little
- ○ A moderate amount
- ○ A lot
- ○ A great deal

Q6 (`HH3`) How much do you know about this topic?

- ○ None at all
- ○ A little

- A moderate amount
- A lot
- A great deal

Q7 (RC) Which statement most reflects your belief on the issue of housing:
- "Local laws that limit rent increases (like rent control) have a positive impact on the amount and quality of affordable housing"
- "Local laws that limit rent increases (like rent control) have a negative impact on the amount and quality of affordable housing"

Q8 (IM) Which statement most reflects your belief on the issue of immigration:
- "When an immigrant takes a job it means there is one less job for a U.S. citizen."
- "Immigration expands the economy and can create more jobs for U.S. citizens"

Q9 (TD) Which statement most reflects your belief on the issue of trade deficits:
- "Trade deficits are bad: if we buy more from other countries than we sell, factories here shrink and jobs leave."
- "Trade deficits aren't automatically bad: they can mean cheaper prices and more investment in the U.S."

Q10 (TC) Which statement most reflects your belief on the issue of taxes:
- "Tax cuts pay for themselves: lower taxes spark more work and investment, the economy grows, and tax revenue rises."
- "Tax cuts don't pay for themselves: any growth bump is usually too small, so revenues fall and the budget hole gets bigger."

Q11 (BA) Which statement most reflects your belief on the issue of trade:
- "Buying American-made products keeps money and jobs in the US, strengthening U.S. businesses and making the country wealthier."
- "Buying American-made products doesn't make us wealthier; if it costs more, we get fewer goods for our money."

3. Smartphone attitude

Q12 (iphonepre) Indicate if you agree or disagree with the following statement.
"Compared to Android phones, Apple's iPhone is worth the additional cost."
- Strongly disagree
- Somewhat disagree
- Neither agree nor disagree
- Somewhat agree
- Strongly agree

4. AI use and trust

Screen Now, answer a few basic questions about AI and then about yourself.

Q13 (ai_use) How often do you use artificial intelligence tools like Chat GPT?

- Have never used it
- Have tried it, but don't use it anymore
- Monthly
- Weekly
- Daily
- Hourly

Q14 (`AItrust_pre`) How much do you trust artificial intelligence chatbots, like ChatGPT, to give you reliable information on topics you care about?

- Completely trust
- Mostly trust
- Somewhat trust
- Mostly distrust
- Completely distrust

5. Demographics

Q15 (`age`) What is your age?
[Open text entry.]

Q16 (`ed`) What is the highest level of school you have completed or the highest degree you have received?

- Less than high school degree
- High school graduate (high school diploma or equivalent including GED)
- Some college but no degree
- Associate degree in college (2-year)
- Bachelor's degree in college (4-year)
- Master's degree
- Doctoral degree
- Professional degree (JD, MD)

Q17 (`race`) Choose one or more races that you consider yourself to be:
[Multiple selections allowed.]

- White
- Black or African American
- American Indian or Alaska Native
- Asian
- Native Hawaiian or Pacific Islander
- Hispanic/Latino
- Other

Q18 (`sex`) What is your sex?

- Male
- Female

Q19 (`income`) **Information about income is very important to understand. Would you please give your best guess?**

Please indicate the answer that includes your entire household income in 2025 before taxes.

- ○ Less than $10,000
- ○ $10,000 to $19,999
- ○ $20,000 to $29,999
- ○ $30,000 to $39,999
- ○ $40,000 to $49,999
- ○ $50,000 to $59,999
- ○ $60,000 to $69,999
- ○ $70,000 to $79,999
- ○ $80,000 to $89,999
- ○ $90,000 to $99,999
- ○ $100,000 to $149,999
- ○ $150,000 or $200,000 *[sic]*
- ○ $200,000 or more

Q20 (`employed`) Are you currently employed?

- ○ Yes
- ○ No, I'm retired
- ○ No, I'm a student
- ○ No, I'm a stay at home parent
- ○ No, I'm looking for work

6. Political identity and ideology

Q21 (`vote2024`) Did you vote in the 2024 Presidential election?

- ○ Yes, for Harris
- ○ Yes, for Trump
- ○ Yes, for another candidate
- ○ No, I did not vote
- ○ No, I can't vote

Q22 (`Q2`) Generally speaking, do you usually think of yourself as a Republican, a Democrat, an Independent, or something else?

[The first three options appeared in random order; "Other" and "No preference" were always last.]

- ○ Republican
- ○ Democrat
- ○ Independent
- ○ Other
- ○ No preference

Q23 (`Q3`) Would you call yourself a strong Republican or a not very strong Republican?
[Shown if "Republican" was selected.]
- ○ Strong
- ○ Not very strong

Q24 (`Q4`) Would you call yourself a strong Democrat or a not very strong Democrat?
[Shown if "Democrat" was selected.]
- ○ Strong
- ○ Not very strong

Q25 (`Q5`) If you had to choose, do you think of yourself as closer to the Republican or Democratic party?
[Shown if "Independent," "Other," or "No preference" was selected. Respondents' answers to this item (or to the party identification item) determined the party and out-party piped into later items and the partisan treatment screens.]
- ○ Republican
- ○ Democratic

Q26 (`social`) On social issues such as same-sex marriage and abortion, where would you place your political views on the following scale?
- ○ 1 - Extremely liberal
- ○ 2 - Liberal
- ○ 3 - Slightly liberal
- ○ 4 - Moderate
- ○ 5 - Slightly conservative
- ○ 6 - Conservative
- ○ 7 - Extremely Conservative

Q27 (`economic`) On economic issues such as taxes and government spending, where would you place your political views on the following scale?
- ○ 1 - Extremely liberal
- ○ 2 - Liberal
- ○ 3 - Slightly liberal
- ○ 4 - Moderate
- ○ 5 - Slightly conservative
- ○ 6 - Conservative
- ○ 7 - Extremely Conservative

7. Affective polarization

Screen Please, indicate if you agree or disagree with the following statements.
[The three items below appeared in random order. Bracketed terms were piped from party identification: the first term was shown to Democrats and the second to Republicans.]

Q28 (`af1`) My identity as a *[Democrat/Republican]* is connected to my core moral beliefs.

- Strongly disagree
- Somewhat disagree
- Neither agree nor disagree
- Somewhat agree
- Strongly agree

Q29 (`af2`) If I found out a friend of mine was a *[Republican/Democrat]*, I would want to stop spending time with them

- Strongly disagree
- Somewhat disagree
- Neither agree nor disagree
- Somewhat agree
- Strongly agree

Q30 (`af3`) *[Republicans/Democrats]* act in ways that us *[Democrats/Republicans]* could never understand

- Strongly disagree
- Somewhat disagree
- Neither agree nor disagree
- Somewhat agree
- Strongly agree

8. Topic assignment and topic introduction

[Not shown to respondents: the survey identified each topic for which the respondent selected the misconception statement and reported at least "A moderate amount" of agreement, and selected one such topic at random. Respondents saw the introduction screen for their assigned topic. Respondents who held none of the six misconceptions discussed smartphones instead and are not part of the analytic sample.]

Screen Next: Brief Chat on Government Budgets

We would like to know more about your thoughts on government budgets.

Please click **Next** to talk to ChatGPT about the topic. *[Shown if assigned topic: government budgets.]*

Screen Next: Brief Chat on Rent Control

We would like to know more about your thoughts on rent control.

Please click **Next** to talk to ChatGPT about the topic. *[Shown if assigned topic: rent control.]*

Screen Next: Brief Chat on Immigration

We would like to know more about your thoughts on immigration and the economy.

Please click **Next** to talk to ChatGPT about the topic. *[Shown if assigned topic: immigration.]*

Screen Next: Brief Chat on Trade Deficits

We would like to know more about your thoughts on trade deficits and the economy.

Please click **Next** to talk to ChatGPT about the topic. *[Shown if assigned topic: trade deficits.]*

Screen **Next: Brief Chat on Tax Cuts**

We would like to know more about your thoughts on taxes and the economy.

Please click **Next** to talk to ChatGPT about the topic. *[Shown if assigned topic: tax cuts.]*

Screen **Next: Brief Chat on Trade**

We would like to know more about your thoughts on buying American products and the economy.

Please click **Next** to talk to ChatGPT about the topic. *[Shown if assigned topic: Buy American.]*

Screen **Next: Brief Chat on Smart Phones**

We would like to know more about your thoughts on smart phones.

Please click **Next** to talk to ChatGPT about the topic. *[Shown to respondents with no eligible topic.]*

9. Bias information (treatment)

[Respondents were randomly assigned with equal probability to one of four screens: no information (control), undirected bias, light out-party bias, or heavy out-party bias. In the two out-party conditions, Republicans (including independents closer to the Republican Party) saw the warning of left-wing bias, and Democrats (including independents closer to the Democratic Party) saw the warning of right-wing bias. The verbatim text of every treatment screen appears in SI Section G.]

10. Conversation with ChatGPT

[Model settings and the system prompt are reported in SI Section E. The conversation had three rounds; each round began with the waiting screen below while the survey retrieved the model's reply.]

Screen We will now forward your answers to ChatGPT

Please wait... Talking with ChatGPT *[Before round 1.]*

Q31 (`human1`) Response from ChatGPT

[ChatGPT's response]

Please write your response below.

[Round 1. Open text box; at least 50 characters required.]

Screen Please wait... Talking with ChatGPT *[Before round 2.]*

Q32 (`human2`) Response from ChatGPT

[ChatGPT's response]

Please write your response below.
[Round 2. Open text box; at least 5 characters required.]

Screen Please wait... Talking with ChatGPT *[Before round 3.]*

Q33 (`Q13`) Response from ChatGPT
[ChatGPT's response]
Please write your response below.
[Round 3. Open text box; response optional.]

11. Posttreatment measures

Screen Please click to the next screen to answer a few more questions.

[Respondents answered the forced-choice item and the agreement item for their assigned topic again. Wording and response options were identical to the pretreatment items (statement order again randomized), with one exception: the first trade-deficit statement read as follows.]

- "Trade deficits are bad: if we buy more from other countries than we sell, factories here shrink, and jobs leave."

Q34 (`howper`) Recall that you had a conversation with an LLM.
How Persuasive did you find the arguments the LLM presented?

- Not at all persuasive
- Slightly persuasive
- Moderately persuasive
- Very persuasive
- Extremely persuasive

Q35 (`use_AI_again`) In the future, how likely is it that you will use ChatGPT to challenge your opinions on economic and political issues?

- Extremely unlikely
- Somewhat unlikely
- Neither likely nor unlikely
- Somewhat likely
- Extremely likely

Q36 (`manipcheck`) Which of the following corresponds to your beliefs about the political bias of ChatGPT
[The five bias options were displayed in either the order shown or the reverse order, at random; "I don't know" was always last.]

- ChatGPT has a strong left-wing bias
- ChatGPT has a weak left-wing bias
- ChatGPT is relatively neutral
- ChatGPT has a weak right-wing bias
- ChatGPT has a strong right-wing bias
- I don't know

Screen Now on to a few more basic questions...

Q37 (`AItrust_post`) How much do you trust artificial intelligence chatbots, like ChatGPT, to give you reliable information on topics you care about?

- ○ Completely trust
- ○ Mostly trust
- ○ Somewhat trust
- ○ Mostly distrust
- ○ Completely distrust

Q38 (`replaceAI`) To what extent do you worry that AI technology could take over your job responsibilities within the next decade?

- ○ Not at all worried
- ○ Slightly worried
- ○ Somewhat worried
- ○ Moderately worried
- ○ Quite worried
- ○ Extremely worried

Q39 (`AIpolicy`) To what extent should policymakers (e.g. Members of Congress) use AI tools when seeking information on policy areas outside their expertise?

- ○ Not at all
- ○ A little
- ○ A moderate amount
- ○ Quite a bit
- ○ A great deal

Q40 (`recall`) Above, we mentioned that Large language models (LLMs) rely on their training data to form responses.

We may have mentioned that there was a "bias" or "skew" in the training data.

Please select the correct "bias" we mentioned.

[Options are listed in display order: the first option was always shown first; the remaining five appeared in random order.]

- ○ We didn't mention a specific skew or bias
- ○ Left-wing or liberal skew
- ○ Right-wing or conservative skew
- ○ A Western (US and Europe) bias
- ○ An English language bias
- ○ A historical bias toward the internet era

Q41 (`othersurvey`) Have you ever taken a survey where you have had to talk with ChatGPT before?

- ○ Yes
- ○ No
- ○ Not Sure

Q42 `(javascript)` *[Optional coding task; open text box.]*

Optional Coding Challenge

No pressure: We're interested in coding skills. If you don't know how to complete this task or don't want to spend time on it, type **I don't know** in the response box below. That's a perfectly valid answer.

If you're comfortable with JavaScript and want to attempt the challenge, follow the instructions below.

Task specifications:

Write a JavaScript function called `filterAndSum` that takes an array of objects. Each object has:

- `name` (string)
- `value` (number)

The function should:

- Filter to only objects where `name` contains the letter `e`
- Sum the `value` of those filtered objects
- Return the sum

Write your complete JavaScript function in the box below.

Remember: *Most respondents choose to skip coding questions. Typing* ***I don't know*** *will not affect your compensation or standing.*

Screen *[Debriefing.]*

Your AI Conversation Experience

What really happened: In this experiment, you engaged in a conversation with an AI language model (LLM). We were studying how effectively AI systems can present economic perspectives that might differ from your initial viewpoints.

The AI was specifically instructed to:

- Respectfully challenge your initial economic beliefs
- Present alternative perspectives based on current economic thinking
- Remain truthful and accurate in its responses

Important note about AI bias: *You may have seen information suggesting the AI's training data was politically skewed. While concerns about AI bias exist in public discussions, research on the exact nature and direction of these biases is still ongoing and inconclusive.*

Thank you for participating in this research on human-AI interaction!

References

Aigner, Dennis J. (1973). Regression with a binary independent variable subject to errors of observation. *Journal of Econometrics 1*(1), 49–59.

Bisbee, James and Arthur Spirling (2026). What to do when humans are no longer the gold standard: Large language models, state of the art and robustness for politics research. Working paper, May 2026 draft.

Blair, Graeme , Alexander Coppock, and Macartan Humphreys (2023). *Research design in the social sciences: declaration, diagnosis, and redesign.* Princeton University Press.

DiGiuseppe, Matthew and Michael Flynn (2026). Scaling open-ended survey responses using LLM-paired comparisons. *Public Opinion Quarterly*. Forthcoming.

Wang, Peiyi , Lei Li, Liang Chen, Zefan Cai, Dawei Zhu, Binghuai Lin, Yunbo Cao, Qi Liu, Tianyu Liu, and Zhifang Sui (2024). Large language models are not fair evaluators. In *Proceedings of the 62nd Annual Meeting of the Association for Computational Linguistics (Volume 1: Long Papers).*

Zheng, Lianmin , Wei-Lin Chiang, Ying Sheng, Siyuan Zhuang, Zhanghao Wu, Yonghao Zhuang, Zi Lin, Zhuohan Li, Dacheng Li, Eric P. Xing, Hao Zhang, Joseph E. Gonzalez, and Ion Stoica (2023). Judging LLM-as-a-judge with MT-bench and chatbot arena. In *Advances in Neural Information Processing Systems 36 (NeurIPS 2023).*
